# A Model-Based Analysis of Chemical and Temporal Patterns of Cuticular Hydrocarbons in Male *Drosophila melanogaster*

**DOI:** 10.1371/journal.pone.0000962

**Published:** 2007-09-26

**Authors:** Clement Kent, Reza Azanchi, Ben Smith, Adrienne Chu, Joel Levine

**Affiliations:** Department of Biology, University of Toronto at Mississauga, Mississauga, Ontario, Canada; University of Exeter, United Kingdom

## Abstract

Drosophila Cuticular Hydrocarbons (CH) influence courtship behaviour, mating, aggregation, oviposition, and resistance to desiccation. We measured levels of 24 different CH compounds of individual male *D. melanogaster* hourly under a variety of environmental (LD/DD) conditions. Using a model-based analysis of CH variation, we developed an improved normalization method for CH data, and show that CH compounds have reproducible cyclic within-day temporal patterns of expression which differ between LD and DD conditions. Multivariate clustering of expression patterns identified 5 clusters of co-expressed compounds with common chemical characteristics. Turnover rate estimates suggest CH production may be a significant metabolic cost. Male cuticular hydrocarbon expression is a dynamic trait influenced by light and time of day; since abundant hydrocarbons affect male sexual behavior, males may present different pheromonal profiles at different times and under different conditions.

## Introduction

Chemical communication is fundamentally important to the biology of many organisms. For example, several reproductive behaviors are mediated by chemical signals in insects[1]. Research on chemosensory function has advanced beyond the initial identification of olfactory and gustatory receptors to include mechanisms involved in the production, emission, and neural processing of chemical signals [2], [3].

Chemical signals emitted by *Drosophila* are made within the fly and are found on the body surface, and include sex pheromones [4]–[6]. Courtship between flies is modulated by these compounds. Other social interactions in *Drosophila*, such as the social resetting of circadian clocks [7], are also mediated by chemical cues. The analysis of such chemical signals has been complicated, in part by the high variability between flies.

The genetics associated with the metabolism of chemical signals in *Drosophila* provides a glimpse of natural history. This insight arises from several points: genes responsible for key enzymes in cuticular hydrocarbon (CH) synthesis have been isolated [8]–[12], mutations or natural variants of these genes have been shown to change CH levels within a species and this system contributes to reproductive isolation between sibling species such as *D.melanogaster*, *D. simulans, D. santomea,* and *D. sechellia* [12]–[17]. In addition, the balance between different CH compounds within a single genetically uniform strain of *D. melanogaster* is changed by environmental conditions such as rearing temperature [18]. Thus, an objective of this study is to understand quantitatively how the cuticular hydrocarbon phenotype of *D. melanogaster* varies in response to environmental variables and to the passage of time. Such insights contribute to our understanding of the phenotypic plasticity of this important trait.

Here we use wild-type males to determine (a) the natural range of variation of CH expression of a single genotype under controlled environmental conditions, (b) whether there are time of day patterns in such variation, (c) where patterns of expression co-vary between compounds and what this reveals about chemical pathways of CH synthesis, and (d) what natural variation in CH levels implies about the metabolic cost of pheromone signaling.

In order to study daily variation in these compounds we have had to review methods for analyzing these compounds. We have characterized these methods and identified an underlying general linear model of cuticular hydrocarbon abundances which explains a higher percent of variance than previous methods. The model can be used to deduce new features of CH variation, but perhaps its most important application is to a normalization method which considerably reduces error variances in CH measurements.

Using our model-based analysis, we show that there are five clusters of compounds whose abundance patterns co-vary, and that membership of these clusters is a function of chemical structure and chain length. This demonstrates that although CH abundance variation is high, it is nonetheless strongly constrained by chemistry. We find significant differences in the patterns of hydrocarbon abundance in these clusters in LD (12 hours light-12 hours dark) and in DD (constant darkness), significant differences for two clusters in day versus night abundance, and highly significant cyclical components in the 24 hour variation whose frequencies vary between clusters and between LD and DD. We estimate hydrocarbon turnover rates on the cuticle, leading to the conclusion that chemical signaling has a significant metabolic cost for male flies.

We also identify a new feature of CH variation we call Abundance Variability (AV) by analogy to the beta volatility term of mathematical portfolio analysis [19]. AV is not a chemical volatility; it represents the magnitude of an individual compound's response to fluctuations in CH total abundance (TA), and varies independently of mean compound abundances over time and in response to light. AV is strongly influenced by cluster membership, carbon chain length, and double bond position, further demonstrating that the underlying chemistry of CH production constrains the observed patterns of CH abundance. These findings reveal complex features of chemical physiology and behaviour.

## Results

We consider first the distribution of total CH abundance in individual flies and how this affects different classes of compounds. From this we derive an unbiased normalization technique for CH abundances and use this to characterize patterns of CH coexpression. Clustering methods applied to such normalized data reveal clusters of coexpressed compounds which are related to the chemical synthesis pathways of the compounds. We show that cluster membership correlates with phenotypes such as day-night mean compound differences and effect of light on abundance patterns. Finally we show that frequencies of temporal differences in hydrocarbon abundance span a range from 24 to 3 or less hours, and use this to estimate minimum CH turnover rates per day.

### Patterns of Total and Relative Abundance

Patterns of abundance of CHs might be unique to particular genotypes or environmental conditions, or they might be constrained by the underlying biochemical synthesis pathways to a sex- and species-specific program. *D. melanogaster* total CH abundance is highly variable even among genetically identical male flies in the same environmental conditions (over 4-fold variation). High endogenous variation within a condition can obscure between-condition changes.

**Figure 1:**
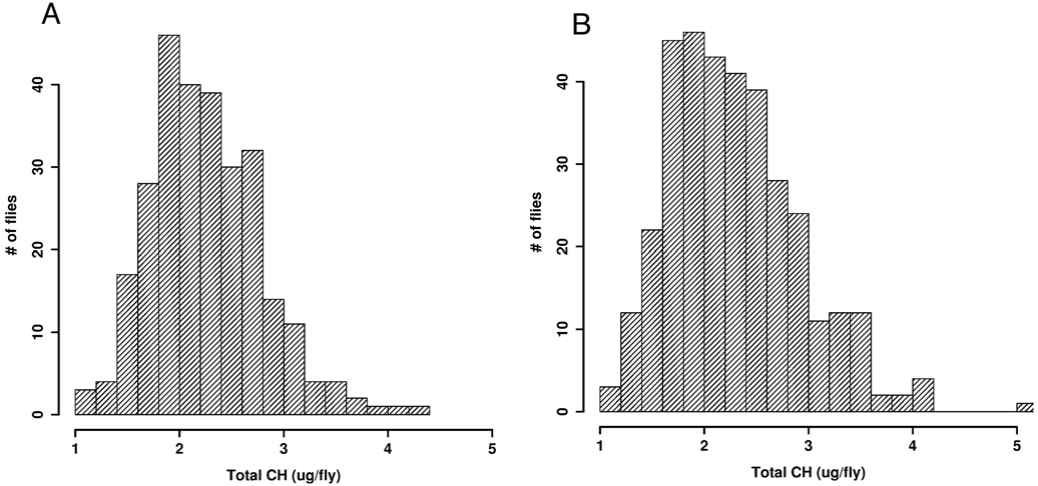
Distribution of individual fly total abundance (TA) values for wild-type males. (A) LD and (B) DD. Mean, LD = 2.27 µg/ fly (N = 277), DD = 2.30 µg/fly (N = 348.); no significant difference. The standard deviation in DD is 23% higher than in LD (F_347,276_ = 1.518, p = .00015).

The pattern in total CH abundance (TA) is illustrated in figure 1, showing the very wide spread of individual fly values. The distribution of total abundance is approximately lognormal; it is not uncommon to find flies in the same vial whose total abundance differs by a factor of 2. However, although individual fly TA varies greatly within a vial or condition, the population distribution of TA values observed is reproducible within one condition. Mean TA does not vary with light environment, but the standard deviation of TA is 23% higher in DD (p = .00015) (Figure 1).

No significant temporal pattern in mean TA was detected in either light environment via several techniques, including ANOVA and Fourier decomposition (see Methods). Thus mean TA is insensitive to time and light in our conditions, but individual fly TA varies over a four-fold range within samples. When analyzing abundances of individual compounds, this high TA range causes large error variances.

**Figure 2:**
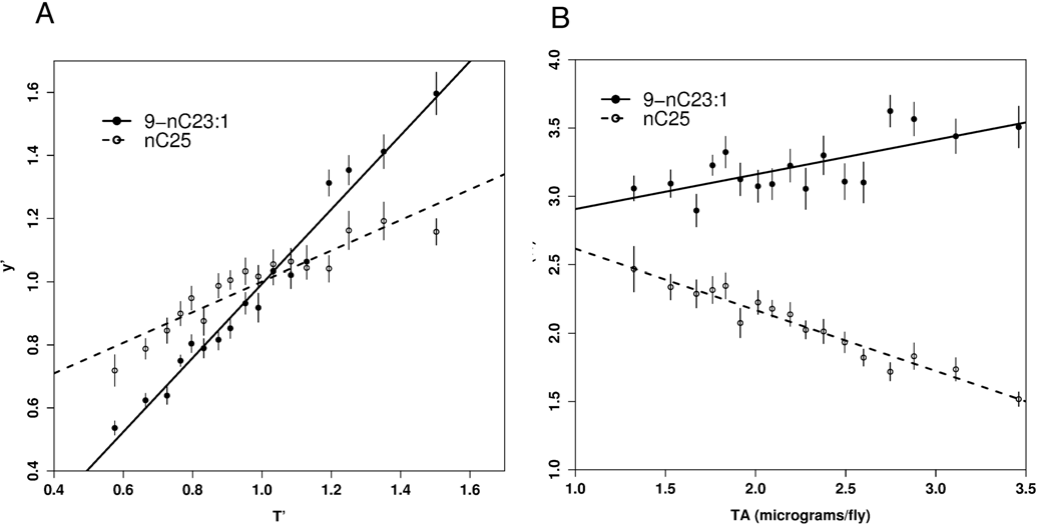
Test of RA model assumptions. (a) Plot of scaled abundance *y*′*_i_*_,*j*_ versus scaled total abundance *T′_i_* for monoene 9-C23∶1 (solid line) and n-alkane C25 (dashed line) in DD. Fitted slopes β′*_j_* are different from 1: slope β′*_j_* = 1.254 for 9-C23∶1 (s.e. = 0.0345, t = 7.37, df = 346, p<1e-12) and β′*_j_* = 0.483 for C25 (s.e. = 0.0365, t = −13.6, df = 345, p<1e-16). Each point is mean±1 s.e. of 20–22 observations over 24 hours. Abscissa and ordinate are dimensionless.(b) Plot of RA versus TA over 24 hours. RA is dimensionless, TA is µg/fly. 9-C23∶1 – filled circles, solid line; C25 open circles, dashed line. RA for C25 is less in flies with high total hydrocarbons, while RA for 9-C23∶1 is higher in such flies.

Several normalization techniques have been used to minimize effects of this high within-treatment endogenous variation. If endogenous variation causes all compounds to vary as a simple multiple of the TA, then expressing each compound amount as a proportion of the TA for the sample is an unbiased estimator of relative abundances (RA). The relative abundance measure also corrects for internal variability of the measurement system, and has been used extensively in *Drosophila* CH literature [16], [20], [21]. Other authors have used a log-contrast method in which the logarithm of the ratio of a compound of interest to another compound is used [22]–[24]. Both methods work well if all compounds respond as a strict multiple to total abundance changes, that is, as a linear relationship passing through the origin. We tested this assumption by fitting a general linear model not constrained to pass through the origin, relating the abundance y_i,j_ of compound j in fly i to a “latent variable” x_i_:
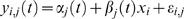
(1)In equation 1, the latent or hidden variable x_i_ represents an overall compound abundance, α and β the intercept and slope, respectively, which depend on time t and compound j, and ε the error or noise term. Latent variable models (factor analysis) are commonly used to test multivariate data for the presence of hidden variables which explain much of the observed variation [25], [26]. Our model in equation 1 is a generalization of the model implicit in the use of the relative abundance measure RA, for if the relative abundance (proportion) of compounds is independent of total abundance, then equation 1 must hold with α_j_(t) = 0 for all compounds j and times t. We call this case the “RA model”. A change of scale helps make clear a prediction of the RA model. If we divide or scale compound and total abundances by their means to get variables y′ and T′ then the RA model predicts (see Methods for details):
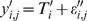
(2)In other words, if we regress scaled compound abundances against scaled total abundance, the intercept α′*_j_* of the regression should not be significantly different from 0 and the slope β′*_j_* should be 1. This assumption fails in our data for most compounds (for 15 of 24 compounds in LD and for 19 of 24 in DD, slope differs from 1 with p<0.05; Figure 2).

When the fitted slope β′*_j_* for compound j is not far from 1, RA will be an acceptable normalization method for reducing error variances. As shown in Figure 2b, the variation in RA values for 9-C23∶1 is 20% of the mean, so in reasonably sized samples standard errors will be small. However, for compounds like C25, the range of RA values is 50% of the mean, leading to larger standard errors.

The good linear fits shown in Figure 2 suggest that Equation 1 can be used to derive a normalization method which incorporates non-zero α*_j_* and slopes different from 1. Below we show how to use the intercept and slope information to more efficiently reduce variation in the data (equation 3). First however we ask if TA is the best latent variable to use. Although total hydrocarbon abundance TA_i_ has a high correlation with compound abundances, there is no *a priori* reason to assume it to be the most efficient estimator of the latent variable x_i_. Factor analysis is used to objectively fit latent variables to linear systems of equations such as Equation (1) (see Methods for details). In the terminology of Factor analysis, values of the latent variable x_i_ are called scores. We explored several methods of estimating scores x_i _(see Methods), and found that total hydrocarbon abundance T_i_ is less efficient than other score estimators.

Given an estimated score x_i_ for a fly, we can use equation 1 to ask what the compound abundances would be if that fly had an average abundance. In other words, we normalize y_i,j_ to its residual value y^N^_i,j_ after the effect of abundance variation is removed. That is, the model-based Factor Analysis (FA) normalization estimates what the CH abundances of a fly would have been, if the fly had an average total abundance:
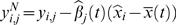
(3)The FA normalization has the important property that it preserves hourly and treatment means and keeps the same units as the non-normalized data (see Methods). Most importantly, the variance of the FA-normalized abundances is much reduced. We calculated the percent of variance removed by normalization for the RA method and FA methods (using several different score estimators) for flies in LD and DD (Table 1).

**Table 1:** Percent of within-hours variance removed by normalization methods.

Compounds	LD–RA	DD–RA	LD–FA	DD–FA
9-C23∶1, 7-C23∶1, 5-C23∶1	73.6%	79.5%	81.3–90.3%	86.1–91.0%
9-C25∶1, 7-C25∶1	53.7%	55.8%	58.6–64.0%	62.9–67.6%
2-MeC24, 2-MeC26, 2-MeC28	33.2%	−5.7% ns	36.7–45.6%	17.7–24.2%
C23, C25, C27, C29	4.7% ns	1.2% ns	32.6–38.4%	31.3–38.2%

RA = Relative Abundance, FA = Factor Analysis. FA values show the range of 3 methods with differing score estimators – see Methods. Values shown are averaged over the compounds indicated. All variance reductions are significant at p<0.01 for each compound in group, except where indicated. ns = not significant. For all compounds and light conditions, the FA normalizations reduced variance significantly more than RA, except for 2-MeC24 and 2-MeC26 in LD.

The factor analysis (FA) normalizations reduced error variances significantly more than the RA technique for all cases except for 2-MeC24 and 2-MeC26 in LD, where the reductions were similar in magnitude. The 3 FA normalizations differ in the methods used to estimate the latent abundance variable x_i_; for details of this and a comparison to other multivariate techniques see Methods.

The slopes β*_j_* in equation 3 depend on the absolute abundance of each compound, which varies greatly between compounds. For purposes of inter-compound comparisons of the slope terms, it is convenient to work with rescaled values β′*_j_* which are independent of compound abundance (see Methods). β′*_j_* will be 1 when compound j increases in identical proportion to changes in abundance variable x – for example, if x increases by 50% and β′*_j_* is 1, the proportional change in abundance of compound j will be 50%. If β′*_j_* is much larger than 1, compound j will be relatively more abundant on flies with high compound abundances – that is, RA_j_ will increase for high abundance flies and decrease in low abundance flies. Conversely, if β′*_j_* is much less than 1 (say β′*_j_* = 0.5; see C25 in Figure 2), an increase of 50% in abundance x_i_ will produce an increase of compound j of only 25%. In this case, RA_j_ will decrease in high abundance flies and increase in low abundance flies.

**Figure 3:**
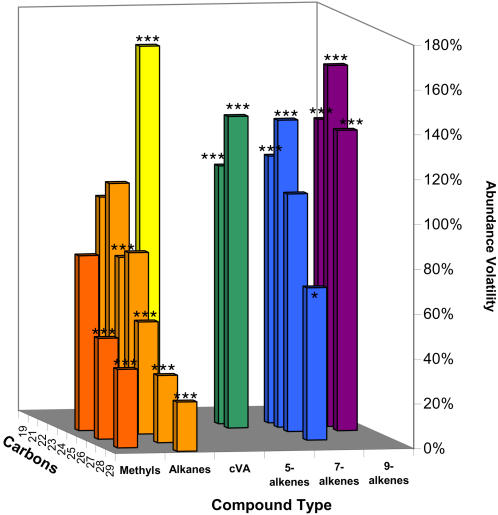
Abundance Variability AV_j_ for 21 compounds in wild-type flies in DD. p(AV*_j_*>0)<10^−7^ in all cases. Methyl-alkanes and n-alkanes have significantly lower AV than monoenes at each chain length (p<10^−6^ in all cases), while longer chain compounds have lower AV than short chain compounds (e.g. AV_C27_ = 0.30<AV_C23_ = 0.74, p<10^−11^). Fitted to response for hours 0–23 using FA latent variable (see Methods). Asterisks indicate significant difference from the null hypothesis of AV = 100%. *** = p<0.001; ** = p<0.01.

Mathematical portfolio analysis calculates a term called beta, which is the ratio between the proportional increase or decrease of a stock and the increase or decrease of the market [19], [27]. This beta statistic is also known as stock volatility, and has the same interpretation as our β′*_j_* – high volatility means the stock increases at a rate faster than the market, low volatility means its response is damped compared to the market. In our case, the abundance of an individual compound is analogous to an individual stock price, and the abundance score x is analogous to the market price. When β′*_j_* is expressed as a percent, we call this the Abundance Variability for compound j, or AV*_j_*. A chart of AV*_j_* values determined for 348 wild-type flies in DD conditions is shown in Figure 3.

Several clear patterns of AV in DD emerge from this analysis. First, monoenes of chain length 23–25 all had AV >100%, while linear alkanes (“n-alkanes”) and methyl-branched alkanes (“methyl-alkanes”) with 23 or more carbons all had AV<100%. Second, within a compound class (e.g. methyl-alkanes, odd-length n-alkanes, even length n-alkanes, 5-alkenes, 7-alkenes, or 9-alkenes), CH with fewer carbons have higher AV values than longer chain compounds of that class. Third, among alkenes the position of the double bond changes AV ranking, with 5-alkene bonds producing lowest AV and 9-alkene bonds the highest. The compound with the highest AV, (cVA, 163%) has the shortest C chain, while the compound with the lowest AV (C29, 22%) has the longest C chain.

In this section we have introduced a general linear model (equation 1) of CH abundance which explains more variance than previous proportion-based models. We derived a normalization method, FA-normalization, from the model which keeps CH data in absolute microgram or nanogram units, does not change hourly or condition means, but significantly reduces within-treatment variance leading to smaller error variances. Just as the linear model fits an intercept and slope, FA-normalization provides both a mean value and a scaled slope, the AV or abundance variability, which varies by types of compound (monoenes higher than n-alkanes) and by carbon chain length and bond position (Fig. 3). We provide a fully worked out example of calculating FA-normalized values and AV in an Excel spreadsheet, with data from hour CT 14 of our DD treatment (see Excel S1).

### Coexpression Clusters parallel chemical pathways

In insects, CH is synthesized in oenocytes, transported in the hemolymph bound to lipophorin, and secreted to the cuticle by processes that are still unclear[1], [28]. Given the intermediate steps between synthesis and deposition on the cuticle, it is not *a priori* clear that observed variation in CH abundance will reflect the underlying chemistry of the compounds. We detect an increasing number of compounds as technical methods become more sensitive, so finding structure among the two dozen or more compounds detected has often relied on statistical analyses using multivariate methods such as Principal Components Analysis[24]. While these methods are powerful, the abstract nature of the patterns found (the component axes) does not always lead to hypotheses about the roles of individual compounds. We used a combination of several multivariate methods to clearly link major components of CH variation to particular groups of compounds in an objective fashion, and to visualize these groups so that position in a chart reflects the main components of variation.

We examine the correlation matrices of (a) FA-normalized compound abundances and (b) FA-normalized β*_j_* values. We fit these independent (a) mean and (b) slope values separately to each hour's data within each treatment, yielding two 24 compound×24 hour matrices of coefficients for each LD/DD treatment, or a total of 4 cases. Using unguided clustering methods (ones in which the algorithm determines where to place clusters, rather than being given predetermined cluster centers) with the Pearson distance measure (the standard way of calculating a dissimilarity or distance from a correlation matrix) we show that compounds that cluster together based on patterns of expression are, for the most part, in the same chemical pathways, but that chain length is also a strong grouping factor.

To simplify viewing the 146 compound correlation pairs for each treatment, we used multidimensional scaling (MDS, synonym for Principal Components Analysis) to create a two-dimensional (x,y) coordinate for each compound, so that compounds with high positive correlations are close together on the plot (see methods for details) [29]. Thus, within each treatment each compound is represented by one point in the figure. MDS scaling is indifferent to sign of the x and y values, so where necessary MDS scalings were reflected and/or rotated to align across treatments – this does not change the similarity distances nor the clustering reported.

To objectively identify clusters of compounds which tend to be coexpressed, we used the non-hierarchical Affinity Propagation (AP) clustering method [30]to identify compounds which cluster together. Lines join compounds in a cluster to a central “exemplar” compound as determined by the AP algorithm (see methods). To assess the dependence of cluster identity on clustering method, we compare the AP results to those from a hierarchical clustering method, Ward's minimum [31].

**Figure 4:**
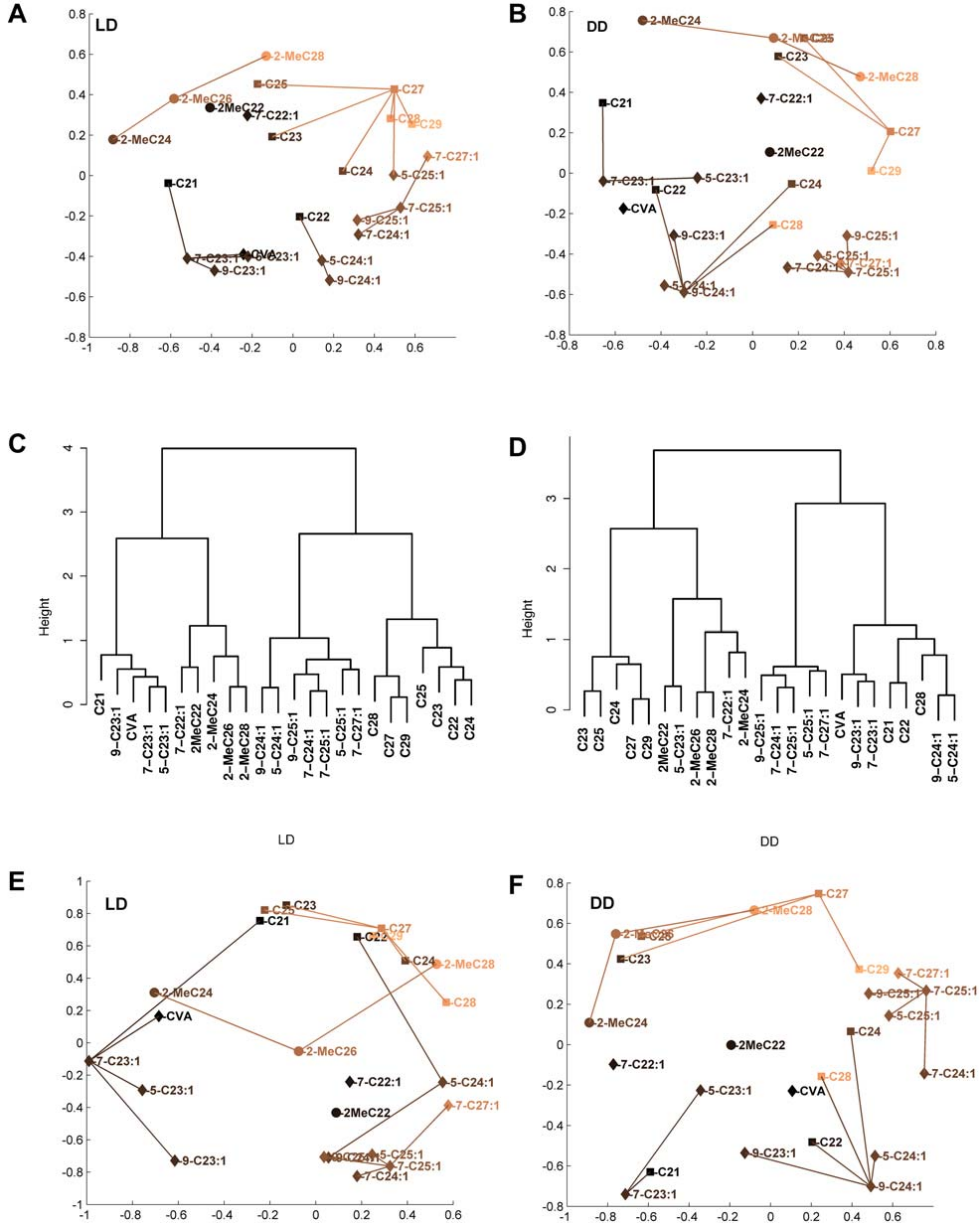
Cluster analyses of 22 CHs in LD and DD. Two independent fitted values per treatment (FA-normalized mean and FA-normalized slope β*_j_*) are used. Clusters based on mean data are shown in A-D, clusters based on slope data, E–F. A,B: MDS/AP clusters of FA-mean data in LD and DD. X and Y axes are multidimensional scaling projections of the 22-dimensional correlation distance matrix into two dimensions. Compounds are joined by a line to a central “exemplar” cluster member if the Affinity Propagation clustering algorithm of Frey and Dueck places them in the same cluster. Different clusters may overlap in this 2-D representation, as two dimensions is not sufficient for MDS projection to capture all of the variation in the data; the AP clusters are determined from the full 22-dimensional structure, which has 4 significant principal components. C,D: Ward's minimum variance clustering of wild-type FA-mean data in DD and LD. E,F: MDS/AP clusters of FA-slope values β*_j_* in LD and DD.

The MDS/AP analysis identifies 5 clusters of compounds whose chemical nature and MDS position are relatively constant (Figure 4, A–F and Table 2) over LD and DD treatments for both FA-normalized means and slopes.

**Table 2:** Cluster Membership.

Compounds	Cluster
C23, C25, **C27**, C29, [*C24, C28*]	1
7-C24∶1, 9-C25∶1,**7-C25∶1**, 5-C25∶1, 7-C27∶1	2
9-C24∶1, **5-C24∶1**, C22, [*C24, C28*]	3
9-C23∶1, **7-C23∶1**, 5-C23∶1, C21, cVA	4
2-MeC24, **2-MeC26**, 2-MeC28	5
2-MeC22, 7-C22∶1	Outliers

Consensus membership of the 5 clusters as determined by the Affinity Propagation algorithm. See also Figure 4 where clusters are shown graphically. Italicized compounds C24 and C28 are assigned to clusters 1 or 3 depending on which data set is used. The core or “exemplar” member of each cluster is shown in bold face.

At upper right of each panel of the MDS/AP figures for FA-mean is n-alkanes Cluster 1, with C27 as the central “exemplar” member. In DD treatments odd chain length n-alkanes only are represented in this cluster, while in LD C24 and C28 are also members. The TA-slope clusters (Fig 4, E and F) are identical to the FA-mean for DD and differ in LD only by the omission of the low-abundance compound 5-C25∶1.

Proceeding clockwise, Cluster 2 is the long-chain alkene cluster, which includes 7-C27∶1, 7-C25∶1, 7-C24∶1, 9-C25∶1, and 5-C25∶1 in three of the four cases (TA-mean DD, TA-slope LD & DD); in the fourth case (TA-mean LD) the only difference is the loss of 5-C25∶1 to Cluster 1 noted above. Interestingly, in all cases treatments 7-C24∶1 is a member of the long-chain alkenes cluster, while its sister compounds 9-C24∶1 and 5-C24∶1 fall into the next cluster.

The third cluster contains core members 9-C24.1 and 5-C24.1 and the even-chain length n-alkane C22 in all four cases. In DD, for both mean and slope datasets, C24, C28 and 9-C23∶1 are Cluster 3 members; in LD C24 and C28 move to Cluster 1 and 9-C23∶1 to Cluster 4.

Cluster 4 is centered on the abundant and behaviorally important compound 7-C23∶1 and has core members 5-C23∶1 and C21 in all 4 cases. In LD it includes 9-C23∶1 and the pheromone cVA (cis-vaccenyl acetate, C_20_H_38_O_2_).

The fifth cluster is specific to the methyl-alkanes 2-MeC24, 2-MeC26, and 2-MeC28. The methyl-alkane 2-MeC22 has high variability due to its low abundance and is not grouped by the AP algorithm.

**Figure 5:**
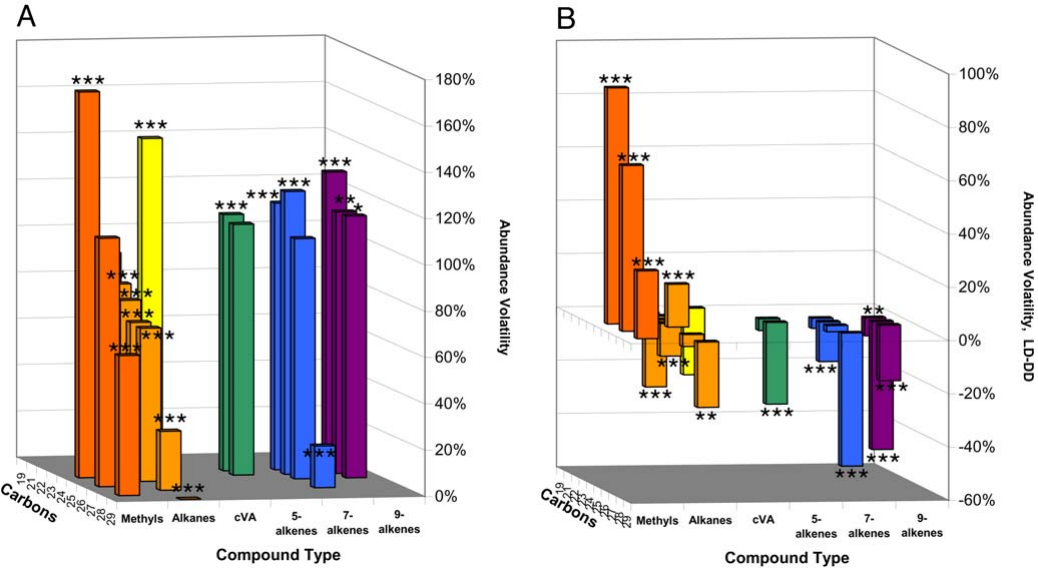
Abundance Variability and effect of light. (A) Abundance Variability AV in wild-type males in LD. (B) Change in AV between LD and DD. *** = p<0.001; ** = p<0.01. Benjamini-Hochsberg FDR q = 0.01, N = 277. Fitted to response for hours 0–23 using FA latent variable (see Methods). Asterisks indicate significant difference from the null hypothesis of (A) AV = 100%, (B) AV LD = AV DD. See Figure 3 for AV in DD.

When we look at the two MDS axes for TA-mean data (Fig 5, A and B) by chemical cluster, we see that the primary (x) axis generally correlates with chain length, while the MDS y axis separates monoenes from methyl-alkanes and long-chain n-alkanes. In addition, considered as a group, the mean y-axis position of odd-chain length n-alkanes is greater than the mean position of even chain n-alkanes, and on average tricosenes and pentacosenes are above tetracosenes. Thus, the y-axis may be viewed as a bond-type and chain parity discriminator. For TA-slope data, the MDS y-axis still separates n-alkanes from monoenes, but the methyl-alkanes cluster is anomalously placed in LD (see below, Figure 5 B, for changes in abundance variability AV_j_ between LD and DD).

The alkene 7-C22∶1 is relatively abundant, making up 10–11% of total CH abundance or 15–16% of total monoenes. Relative abundance of 7-C22∶1 is negatively correlated with most compounds in each of the treatments. Conversely 2-MeC22 and 5-C25∶1 were frequently below detection limits and thus are “noisier” data. Neither 7-C22∶1 nor 2-MeC22 is placed in clusters by AP clustering except in TA-slopes in LD (Fig. 4 E) where they appear in their own “outliers” cluster.

The Ward's hierarchical clustering method applied to TA-mean datasets generally paralleled the non-hierarchical AP method, if we examine branches of the hierarchy at a depth of two or three from the root. For example, in DD Ward's has a branch containing C23,C25, C27 and C29, exactly parallel to the AP Cluster 1; in LD the branch at depth 2 contains all n-alkanes plus 5-C25∶1, the same as AP LD Cluster 1 except for the inclusion of C21 and C22. Methyl-alkanes are clustered together in both LD and DD at depth 3. However, the correspondence between Ward's and AP is more variable for monoenes. Cluster 2 members appear in a single branch at depth 2 in DD, but in LD the depth 2 branch merges members of clusters 2 and 3. For Cluster 4 (core members 7-C23∶1 and 5-C23∶1), in DD Ward's merges the cluster with cluster 2, while in LD Ward's merges Cluster 4 with Cluster 5.

In this section we asked whether each of the two components of our general linear model (equation 1), the FA-normalized values and the AV slopes, would reveal coherent groups of compounds with similar expression patterns, and whether these groups would depend on the LD/DD environment. By looking at FA-normalized data, we subtracted out the obscuring effects of high TA variation. Applied to the normalized data, the MDS/AP clustering technique produces five nearly identical clusters in each of two different datasets (LD and DD) for each of the model's two components (AV and FA-normalized values). The clusters correspond closely to chemical groupings (methyl-alkanes, monoenes) but divide monoenes into 3 groups based on both chain length and double bond position. Abundant odd chain length n-alkanes are consistently placed in the same cluster, but even chain-length n-alkanes may be placed either in Cluster 1 or Cluster 3 depending on LD or DD treatment.

Thus, we have found five coherent compound clusters based on CH abundance patterns which clearly relate to the chemistry of CH compounds. Chemically similar compounds vary together. In the following sections we ask in more detail whether trends in compound abundances due to light or time of day are consistent among cluster members.

### The Effect of Light

Wild-type flies kept in 12 hour light∶12 hour dark (LD) versus continuous dark (DD) conditions were compared to determine whether patterns of CH mean abundance and abundance variability are affected by exposure to light in a conventional photoperiod. In this section we look only at the overall effect of light by comparing 24-hour averages of slope (AV) and mean (FA-normalized values) between LD and DD, reserving details of the interaction of LD/DD and time of day for a later section. Note that our experimental design does not distinguish between direct effects of illumination and indirect effects of LD light cycles synchronizing circadian rhythms.

Figure 5 shows AV, Abundance Variability, for wild-type in LD, and the difference between LD and DD values. 7- and 9-monoenes still have significantly higher AV than n-alkanes in LD, but the abundance variability of methyl-alkanes increases in LD (p<0.0002 after Bonferroni correction). AV decreases in LD for most n-alkanes and alkenes, significantly for, 7-C24∶1, C24, 9-C25∶1, and C29, and highly significantly (p<0.0001) for cVA, C22, 9-C24∶1, 5-C24∶1, 5-C25∶1, 7-C27∶1, and C28. Anomalously among n-alkanes, C25 has a significant increase in AV in LD (p<.0001).

**Figure 6:**
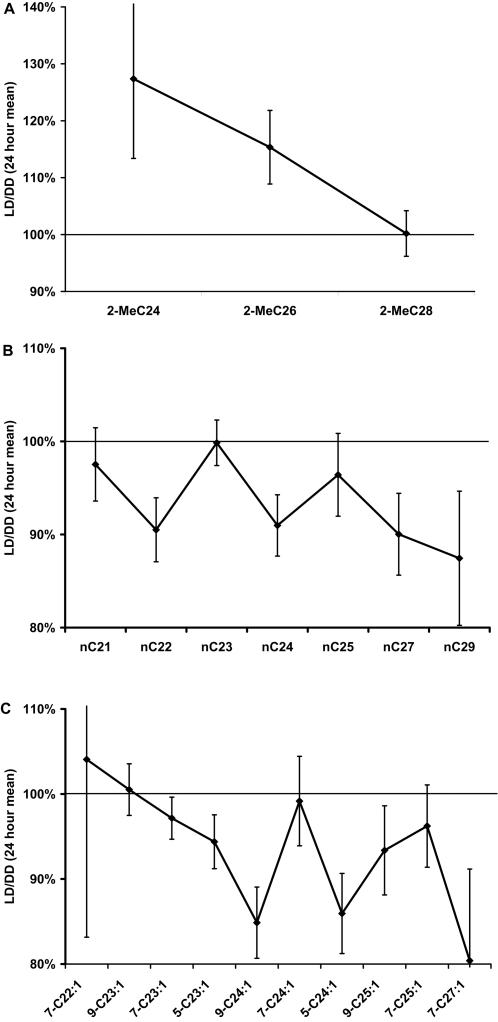
Compound TA-mean abundance ratio, LD/DD. Short chain methyl-alkanes increased in LD, n-alkanes decreased or did not change. Among monoenes, 7-C23∶1 and 9-C25∶1 decreased slightly but significantly in LD (p<0.003), while 5-C23∶1,9-C24∶1,5-C24∶1, and 7-C27∶1 decreased more (p<5e-6). Error bars show 99% confidence intervals. Averages of 24 hours of data were compared for the two treatments.

To show the overall effect of light on each compound, we compared the FA-normalized mean values averaged over 24 hours, between LD and DD. The results (Figure 6) show LD as % DD (100% = no difference) along with the p-value for a t-test. In all cases where we apply tests to multiple compounds in parallel, we use the Benjamini-Hochberg False Discovery Rate (FDR) [32] at a value of q = 1/25. Thus, in tests on 24 compounds, we expect on average 1 or less false discoveries. Our value of p used for confidence limits is set at 0.01, so error bars shown are more stringent than the usual 0.05 level.

The presence of 12 hours of light caused FA-normalized mean abundance of methyl-alkanes to increase significantly for 2-MeC24 and 2-MeC26. The effect of light diminishes as methyl-alkane chain length increases. No n-alkanes or alkenes increased in LD. Decreases were larger for the even chain compounds C22, C24, 9-C24∶1, and 5-C24∶1 (p<1e-11 in each case), but less significant for the odd-chain monoenes 7-C23∶1 (p = 0.003), 5-C23∶1 (p = 5.5e-6), 9-C25∶1 (p = 0.001), and 7-C27∶1 (p = 3.3e-06). Thus, overall daily average FA-normalized compound levels increase in LD over DD for some short chain methyl-alkanes, and decreased for some monoenes and n-alkanes.

### Temporal CH Pattern

In this section we describe which compounds showed diurnal (day versus night) differences for each light treatment, and when (what time of day) differences between LD/DD treatments were most marked. We examine the three levels of variation described previously: total abundance TA, abundance variability AV, and variation of individual compounds. Finally, we look at whether cyclic fluctuations can be found in compound abundance over time, and if so what the periods of such cycles are.

### Total Abundance

No significant effect of hour on log(TA) was found in either LD or DD. Low TA values at hours 6 and 13 produced F tests at the p = .02 level at CT 6 and p = .009 at CT 13, but on applying the FDR control at q = 1/25, none of the individual hourly F tests were significant. A note of caution is required: because of the high variability of TA data and the large number of tests (1 per hour), considerably more than our N = 15 replicates per hour might be required to detect small changes in mean TA. Within the limits of our sample sizes, therefore, the distribution of TA values appears to be independent of time of day.

### Mean Abundance

**Figure 7:**
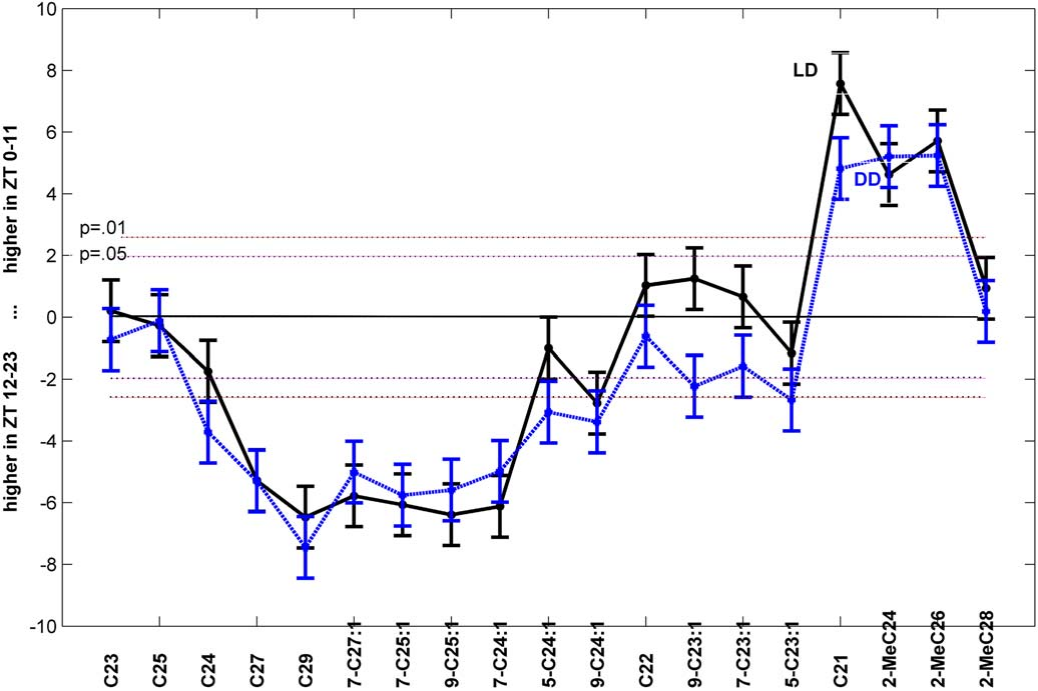
Significance of differences between subjective day and subjective night FA-normalized mean compound abundance in LD (solid line) and DD (dashed line). Y axis gives Student t test value for comparison (df = 275 in LD and 346 in DD for each compound). Error bars, ±1 s.e. Critical values of t for a two-sided test are given for p = .05 and .01. Compounds ordered by cluster membership – see Figure 4 (low abundance compounds are omitted). C21 and shorter chain methyl-alkanes have highly significantly higher mean in day than at night, while long chain n-alkanes (cluster 1) and clusters 2 and 3 (longer chain monoenes) highly significantly higher mean at night. For most compounds, mean day-night difference is not effected by presence of light in day (LD) or its absence (DD). However, cluster 4 members (tricosenes and C21) do show a significant increase in the day-night mean difference in LD.

Perhaps the simplest question which can be asked about temporal variation in compound abundance is whether mean values during subjective day (CT 0–11) differ from means during subjective night (CT 12–23). We plot the Student t value for the comparison of day versus night for a number of compounds, ordered by cluster membership, in Figure 7. Positive t indicates the mean in day exceeds mean at night for that compound. LD and DD t values are shown as two curves; their general parallelism suggests that light itself has little effect on day-night differences, except in Cluster 4 members (tricosenes and others), where presence of light in LD causes a significant increase in the mean day-night difference.

In general, Figure 7 shows that within n-alkanes, methyl-alkanes, and monoenes, longer chain compounds have significantly higher mean values during the night hours than in day, while for short chain compounds, only methyl-alkanes and C21 are significantly higher over the day hours. Furthermore, for day versus night means, only Cluster 4 members respond to light.

**Figure 8:**
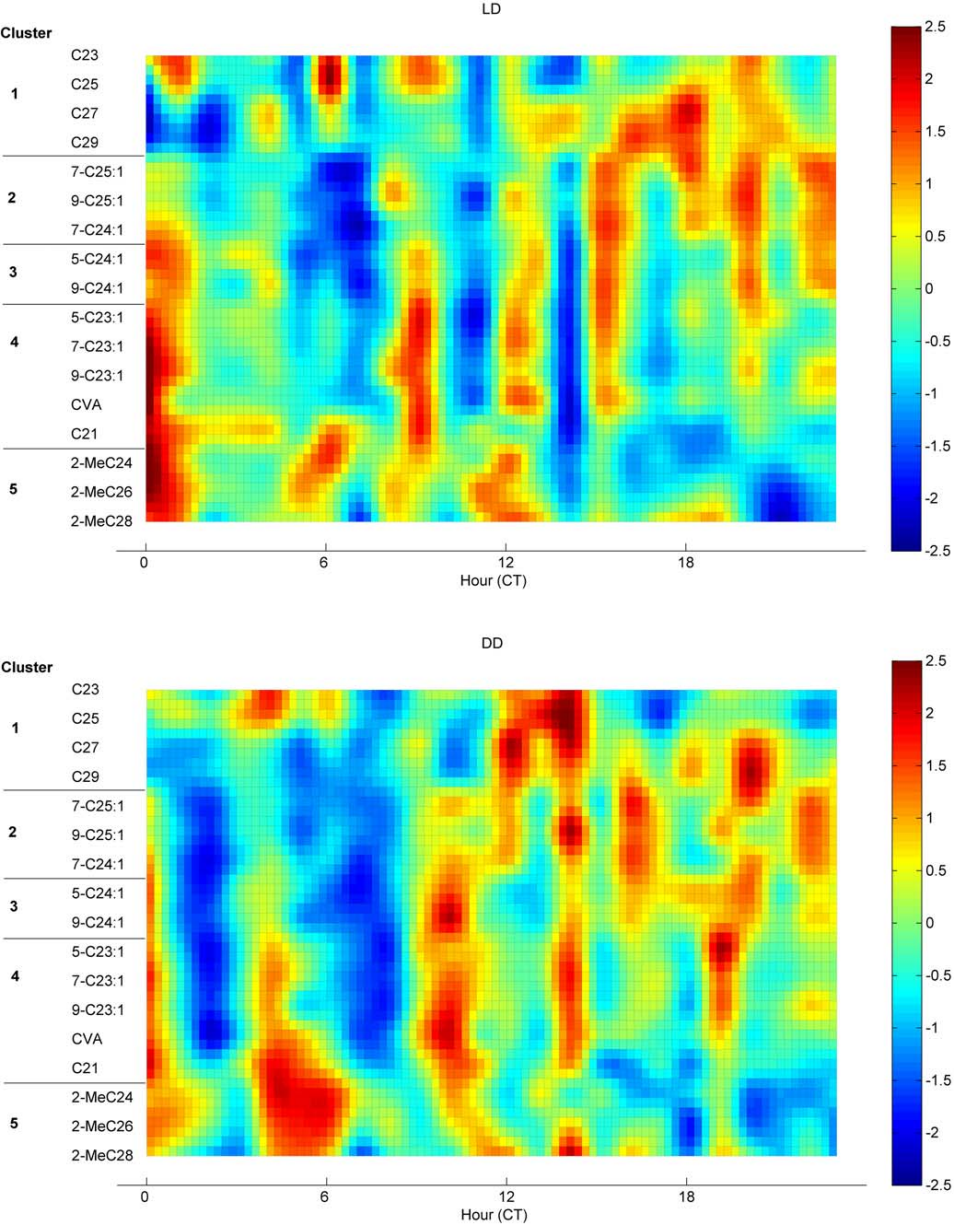
Diurnal changes in compounds. (A) LD (B) DD. Heat map shows compound hourly mean scaled to daily mean of 0 and SD of 1. Units of color map are SD. Note LD minimum at CT 14 becomes a peak for most compounds in DD; similar effects occur around CT 10 in clusters 2–4.

The broad day-night patterns shown above become more complex when viewed on an hourly basis. To summarize the detailed trends in variation from CT 0 through 23, we plot the deviation of hourly FA-normalized abundance from the 24-hour average in Figure 8. A value of 2 indicates the compound is two standard deviations above the daily mean at that hour. We order compounds by their cluster membership in the MDS/AP cluster diagrams, and we omitted several low-abundance compounds that were highly variable (2MeC22, 5-C25∶1, 7-C27∶1, C28).

These heat maps highlight several important points. First, although the day-night differences demonstrated in Figure 7 are visible, there is large variation between hours within day or night, and this variation is usually synchronous among cluster members. Second, the timing of hourly variation is changed by light condition (LD/DD) at CT 10 and 14. In LD, long chain n-alkanes in Cluster 1 and long chain alkenes in cluster 2 have generally lower levels in day and one to several peaks at night, while short chain n-alkanes also show peaks within day hours. Compounds in Clusters 4 (tricosenes, etc) and 5 (methyl-alkanes) have two or three peaks in abundance during the day and fewer peaks during the night. Compounds in Cluster 3 show patterns intermediate between those described above, but still show 2 or more peaks over 24 hours.

**Figure 9:**
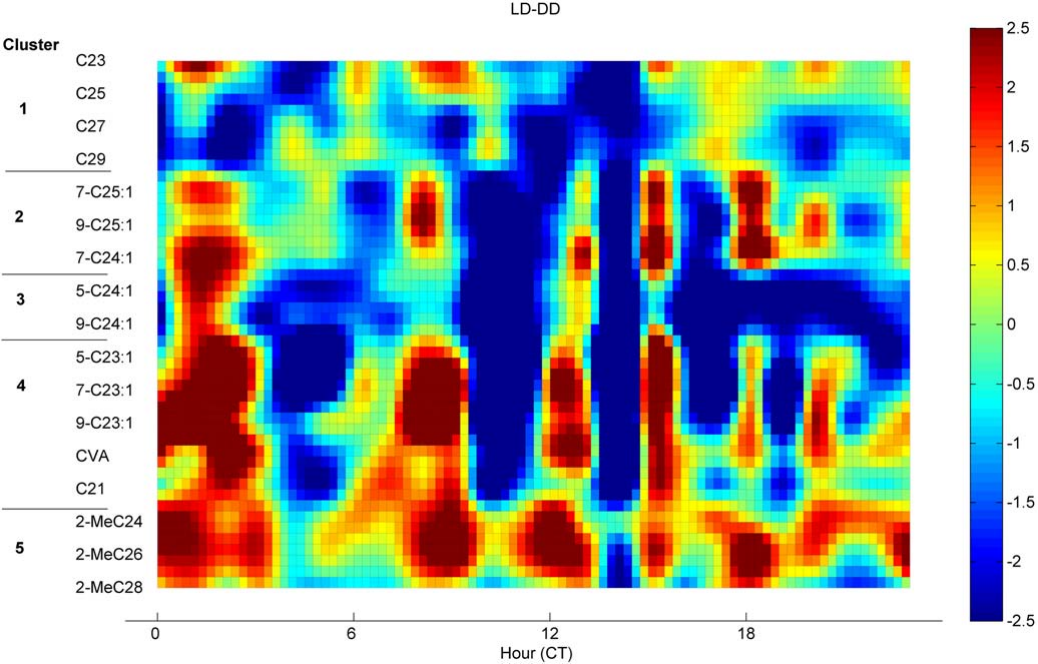
Difference between LD and DD at each hour. Heat map shows t statistic for comparison of FA-normalized compound abundances at each hour; df = 25–28. Dark red areas indicate significant LD excess, dark blue, significant DD excess.

In Figure 8 we analyzed hour-to-hour variation for compounds within LD and DD. We may also ask whether hourly variation patterns change between LD and DD, that is, whether the presence or absence of light interacts with temporal variation. In Figure 9 we show the difference between hourly LD means and hourly DD means, again as a t-statistic heat map where a value of 2.07 (dark red) indicates LD>DD at p<0.025, and a value of −2.07 indicates DD>LD (two sided t-test with df = 23). Cluster 1 (n-alkanes) has lower abundances in LD than DD at a majority of hours, while Cluster 2 (long chain monoenes) also is lower in LD than DD much of the time, but has isolated reversals at times CT 2, 8,15 and 18 where LD>DD. Note that overall the compounds in clusters 1 and 2 tend to be higher at night than in the day (Figure 7), but we can see that there are important exceptions to the overall trend at the hourly level. Cluster 4 and 5, which in Figure 7 tend to be higher overall in day than night, are generally higher in LD than DD, although this trend is sharply reversed at CT 10 and 14. There are 5 periods at which many compounds show significant LD excess (CT 1–2, 7–8, 12–13, 15, 18) and 4 periods during which compounds show significant DD excess (CT 4–5, 10–11, 14, 16–17). These differences are most significant in Cluster 4 but are seen in each of clusters 2–5.

Thus, presence of light during CT 0–11 not only changes overall means in LD versus DD as shown in Figure 7 for Cluster 4 members, it also causes sharply different hourly abundances that oscillate between LD excess and DD excess, with the sharpest, most significant such differences happening between CT 0 and 15 for compounds such as tricosenes and short chain methyl-alkanes. We will return to these sharp LD-DD differences in a later section where we look at the differing cyclic components of temporal pattern in LD and DD.

### Abundance Variability

**Figure 10:**
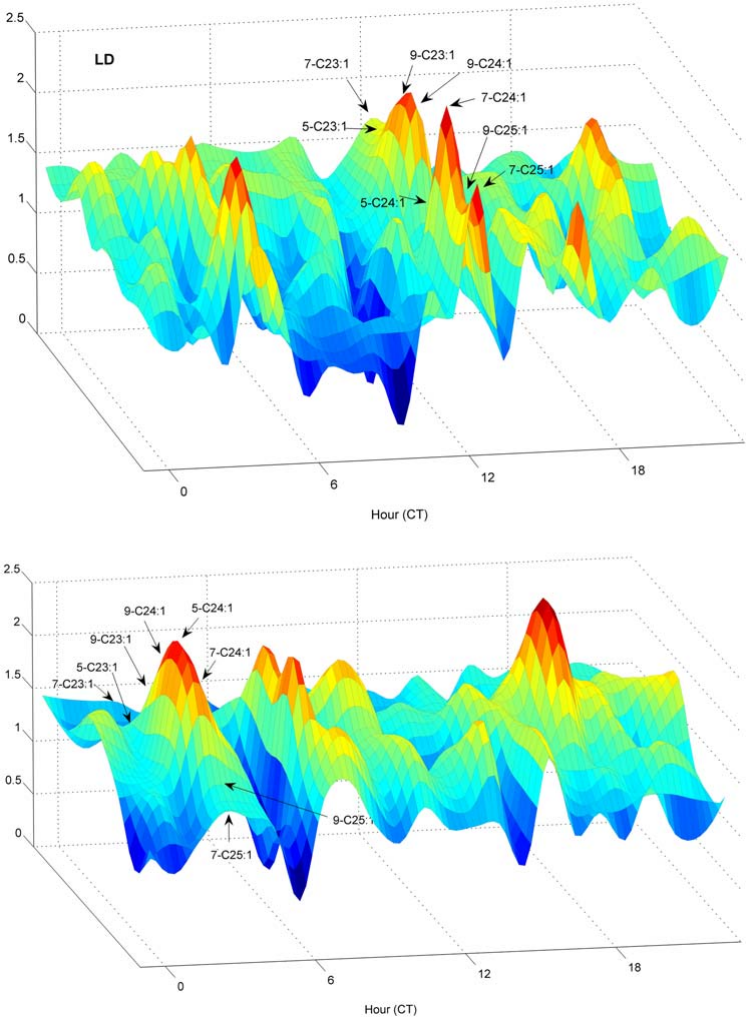
Abundance Variability versus time of day for monoenes. AV values were fitted to 1-hour intervals for flies in (a) LD, (b) DD. A peak of AV at CT 4 and a low at CT 6 occur in both LD and DD, but pentacosene LD peaks are higher than in DD. Note the prominent LD peak at CT 13 for all monoenes, which is absent in DD. Surface shown is hourly data points plus intermediates produced by bicubic interpolation.

Abundance Variability (AV), the measure of how much a compound's absolute abundance increases as total abundance increases, is an independent measure of CH variation from FA-normalized mean abundance. AV also shows significant hourly temporal variation in DD and LD (Figure 10).

**Figure 11:**
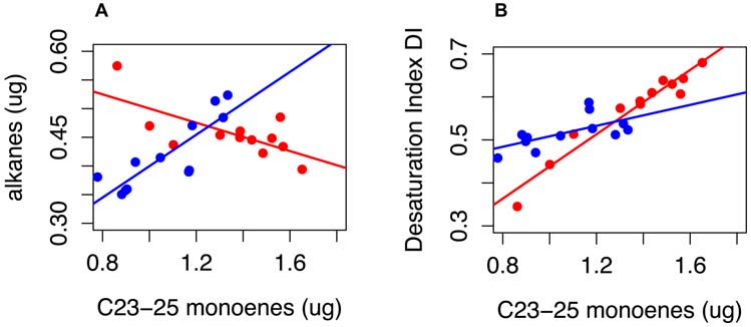
Effect of LD AV variation at CT13. (A) n-alkanes versus monoenes (B) Desaturation Index DI versus monoenes. (A) Sum of n-alkanes versus sum of C23–C25 monoenes, in LD. Non-normalized data is used. Red, CT 13; blue, CT14. Slope for CT13 differs significantly from CT 14 and other hours (p<2e-5) (CT 13, slope −0.12±0.04; CT 14 slope 0.27±0.05; other hours, 0.19±0.01). Compare with Figure 10 (a), AV by hour in LD, where a peak in monoene AV occurs at CT 13 and a minimum at CT 14. (B) The Desaturation Index (DI[33]) calculated for the same flies. Slope for DI versus total monoenes – CT 13: 0.374±.0295; CT 14: 0.122±.0468; other hours: 0.113±.00828. CT 13 slope differs from other hours (p = 5e-06).

In LD, alkenes have a peak of AV at CT13 which is absent in DD, while in DD a peak occurs at CT7 which is absent in LD. In LD, a minimum of n-alkane AV occurs at CT 13 (data not shown). The consequence of the large monoene AV increase at CT 13 in LD is shown in Figure 11, which graphs total n-alkanes per fly versus total C23–C25 monoenes, at both CT 13 (red points and line), and CT 14 (blue points and line). At most hours, there is a highly significant positive correlation between n-alkanes and C23–C25 monoenes (*r* = 0.78, df = 240, p<2.2e-16) but at CT 13 the correlation is negative (*r* = −0.69, df = 10, p<0.012). Thus, the unusual AV peak for monoenes and minimum for n-alkanes at CT13 points to a complete reversal of the normal relationship between n-alkanes and monoenes which holds at most other hours. As shown in Figure 10, this AV excursion peak is restricted to a single hour, and is flanked by strong monoene AV minima at CT 14 and CT10, implying that in as little as 1 hour the correlation between monoene and n-alkane amounts completely reverses. Indeed, at CT 14 the n-alkane-monoene correlation is positive (*r* = 0.847, df = 10, p = .0005).

Several authors have used what Rouault et al. [20] call Balanced Ratio or BR indices to quantify the relationship between alkenes and n-alkanes[18]. These indices span a range from −1 to 1 and encapsulate the balance between high alkenes (+1) and hence high desaturase activity, and low alkenes (−1) with no desaturase activity. One particular instance of a BR index is the Desaturation Index (DI)[33]. Marcillac et al. defined DI as (ΣDesat−ΣLin)/(ΣDesat+ΣLin) (see Methods). DI values effectively showed the difference between normal flies and desaturase mutants. Marcillac et al. report a value of DI in Canton S (our wild-type) males of 0.566. We observe a daily mean value of 0.559±.064, confirming their results.

The range of DI over all flies in our LD data was 0.35–0.69 (n = 277). This large range includes values as low as the mean DI of some of Marcillac et al.'s Excision-Intermediate desaturase lines. Why should the natural variability in DI cover so wide a range when the flies tested are of a single wild-type genotype? The relationship between n-alkanes and monoenes in Figure 11a suggests an answer. In Figure 11b, we calculate DI for the same flies as in Figure 11a, plotting this against total monoenes. At CT 14 and most other hours, there is a significant positive correlation between DI and total monoenes (and between DI and TA, data not shown) but the slope is small and the range of DI values is smaller. At CT 13, however, due to the change in AV values at this time and their effect on the n-alkane monoene relationship demonstrated in Figure 11a, DI has a significantly higher slope than at other hours (p = 5e-6). This results in DI values which, driven by the normal TA variation, cover a much wider range – indeed, the two extreme DI flies in our LD sample (DI = 0.35 and 0.69) occurred at CT 13. Thus, the variation in AV determined from our general linear model fit explains why DI varies so much at one particular time.

**Figure 12:**
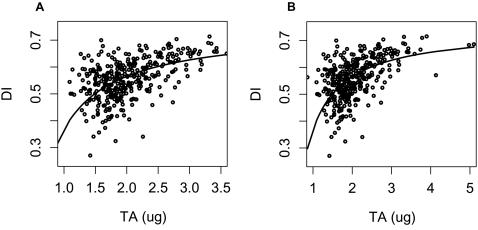
Desaturation Index DI versus total hydrocarbon abundance TA over 24 hours in LD and DD. Line is prediction from fitted multivariate linear model for the compounds used in DI calculations [33]. Predicted and actual DI values are significantly correlated (LD: Pearson's *r* = 0.643, p<2e-16, df = 275; DD: *r* = 0.529, p<2e-16, df = 346).

We have illustrated the relationship between AV and DI with one specific set of data, but applying our model, we may derive a general formula for the dependence of DI (or any other balanced ratio) on overall abundance, in terms of the intercept and slope terms for monoenes and n-alkanes (see Methods for derivation):
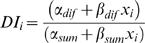
(4)The terms α*_dif_* and α*_sum_* in equation 4 are simply differences or sums of the intercept terms α*_j_* for compounds j in the two groups (alkenes = *Desat* and n-alkanes = *Lin* in Marcillac et al.'s terminology) and the beta terms are similarly differences or sums of compound slopes. At average abundance (***x***_i_ = 0), equation 4 reduces to α_dif_/α_sum_∼0.57 and is similar to Marcillac et al.'s formulation. However, when abundance ***x*** *_i_* is high, DI approaches β_dif_/β_sum_∼0.7, and when ***x*** *_i_* is lower than average, DI declines linearly with ***x*** *_i_*. Within the range of ***x*** *_i_* actually encountered on flies, equation 4 fits very closely to a hyperbolic saturating curve. The hyperbolic dependence of DI on TA is shown in Figure 12 for DD data. The correlation of actual with predicted DI is highly significant (LD: Pearson's r = 0.643, p<2e-16, df = 275; DD: r = 0.529, p<2e-16, df = 346). Thus, flies with higher TA tend to have higher DI, but the relationship is hyperbolic rather than linear.

In this section, we have examined the slope term AV in the general linear model and its changes over time of day and from LD to DD. As with FA-normalized hourly means, AV shows some sharp hourly oscillations of which the most dramatic occurs between CT 12 (lights out, beginning of night) and CT 14, when AV shifts from average, to high, and back to average values. In Figure 11 we saw that the consequence of these AV changes at CT 13 included a reversal of the normal positive correlation between monoenes and n-alkanes, and a very much higher slope of the DI-monoenes relationship. This helped explain why the flies with the most extreme DI values over our 24 hours of sampling were in the CT 13 samples. We then derived a general formula (equation 4) relating DI or any balanced ratio to abundance scores ***x*** *_i_* which is based on simple sums and differences of the intercept and slope terms of the general linear model. In turn, this formula predicts a hyperbolic relationship between DI and ***x*** *_i_* values which is illustrated in Figure 12.

We see therefore that slope or AV values have an important tie to indices such as DI, which in turn relate directly to activity of products of genes such as *desat1, desat2,* and *desatF* [8], [11], [34]. Large hourly changes in AV are related to time in which larger than normal ranges of DI values are observed. We shall return to this point in a later section when we ask how AV changes may be used to estimate CH turnover rates.

### Frequency of CH variation

The frequent changes in hourly FA-normalized compound levels shown in Figure 8 appear to be synchronized over chemical clusters of compounds and to show some regularity. In this section we ask whether there may be cyclic patterns of CH abundances over 24 hours which account for some of these hourly variations.

**Figure 13:**
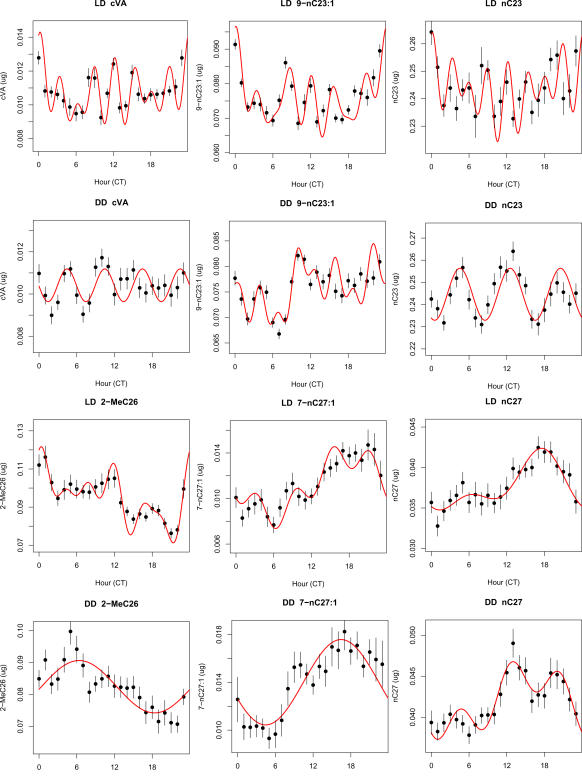
Temporal variation for representative compounds in LD and DD. Curves represent all significant frequencies in a stepwise regression of Fourier components on the raw (unsmoothed) data. Points shown are means from a central moving average filter, ±1 s.e.m. Filter bandwidth is 3, except for curves with significant 3 hour periodicity, where bandwidth of 2 was used (top row). Note 3-hour oscillations centered around peak at lights off (ZT 12) for the three short-chain compounds in LD (significance of 3-hour component for cVA, p_3_<2.3e-10; 9-C23∶1 p_3_<3.3e-05; for C23, p_3_<3.3e-04). In DD, only 9-C23∶1 retains a 3-hour component (p_3_<3.1e-03). For the three C27 compounds in LD the 24 hour period was most significant in LD (7-C27∶1, p_24_<1.0e-07; C27, p_24_<4.7e-05; C27Br, p_24_<4.6e-05) and in DD (7-C27∶1, p_24_<4.5e-07; C27, p_24_<1.2e-05; C27Br, p_24_<2.6e-05). Both the alkene and methyl-alkanes C27 compounds also had significant 6 hour components in LD, but not in DD. All p values derived from F_2,272_ ratios from regression in LD, F_2,341_ in DD (see Methods).

To characterize the temporal pattern of FA-normalized mean abundance, we asked which periods in a stepwise regression of Fourier sine and cosine curves explained significant amounts of variation. In Figure 13 we show the best-fit temporal curves for 3 short chain and 3 longer chain compounds. In LD C23 compounds and cVA had highly significant 3 hour Fourier coefficients, as well as 24 hour terms (9-C23∶1 and C23) or 12 hours (cVA). By contrast, C27 compounds concentrated most of their cyclic variation in 24 hour cycles, as well as 6 hour components. In DD, compounds retained significant 24-hour cycles, but for cVA, C23, 7-C27∶1, and 2-MeC26 there was a reduction in power at short periods compared to LD with no significant 3 hour terms.

We performed the same analyses on RA-normalized as well as FA-normalized data. In LD cyclic terms accounted for 3.3% of total variance in RA-normalized data, versus 8.2% in FA-normalized data. In DD the variances explained by cyclic terms were 6.3% for RA and 16.1% for FA. Thus, FA-normalization more than doubles the ability to detect variation components in data compared to RA; both methods show that in LD cyclic components are on average higher frequency while in DD cyclic variation is twice as large as in LD.

In this section we have shown that cyclic sine and cosine curves with periods dividing 24 hours fit well to the diurnal patterns of compound abundance. High significance of 24-hour periods in both LD and DD suggests an involvement of the circadian clock. However the large amount of variation explained in LD by 3 hour periods for C23 compounds indicates that additional factors beyond the 24-hour clock must be involved. The near disappearance of short period terms in DD strongly implicates light in the induction or maintenance of the high-frequency cycles.

In the next section we shall use the variation explained by cyclic terms as one way to estimate CH turnover rates. Thus, in addition to the intrinsic interest of possible ultradian cycling of CH abundance, there are implications for the metabolic cost of CH signaling.

### Turnover rates for CHs

Levels of CHs vary significantly over 24 hours, so there must be daily turnover of CHs. We use model-based calculations to identify three categories of turnover. First, if the TA level of an individual fly changes during one day over a significant portion of the range of TA values shown in Figure 1, there will be turnover due to TA change. This component of turnover will be proportional to the amplitude of TA variation. Second, if hourly mean levels of a compound change significantly from peak to trough to peak, there will be turnover due to cyclic change. This cyclic turnover will be proportional to peak-trough amplitude times the frequency of cycles, which are shown in Figure 13 to vary from once per 24 hours to once every 3 hours. Third, even if there is no change in TA and hourly compound mean levels in flies, hourly changes in the slope of the relationship between compound abundance and total abundance (AV; see Figures 10 and 11 for hourly change) imply there must be a change in the proportions of different compounds even in a fly with constant TA. This component of turnover will be proportional to the amplitude and frequency of changes in AV.

Turnover due to TA change could be directly estimated were it possible to measure CH levels twice on the same fly; unfortunately our assay is destructive – it kills the fly – so we observe only the population distribution of TA values. As a null hypothesis, we posit that transfer (Gain) of CH from internal stores to the cuticle occurs at constant rate of G µg/day, and that loss from the cuticle is due to more variable events, such as grooming, at a loss rate L proportional to the amount of CH on the cuticle. We may then describe change in TA levels x(t) using a stochastic model:
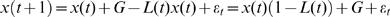
(5)Loss is defined as a random variable *L*(*t*) = θ+ε*_L_* with mean loss rate θ plus a mean zero random component ε_L_, while ε_t_ is a second, independent mean zero random variable. Subject to certain conditions on the distribution of the random variable L, this stochastic process has a well defined stationary distribution whose skewness and variance are related by a term in θ (see Methods); for many cases the distribution is lognormal, as observed in our data (Figure 1). Substituting observed skewness and variance from our data we estimate θ*_LD_* = 0.31 and θ*_DD_* = 0.53. These 31%–53% TA-based turnover values are derived subject to several assumptions and must be regarded as tentative.

**Figure 14:**
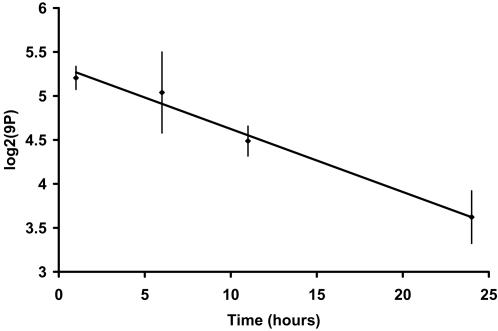
Loss rate of applied 9-C25∶1 in LD. Compound was applied at time 0 (see Methods) and measured on groups of 5 flies at times 1, 6, 11 and 24. Vertical axis shows log_2_(y_t_/y_n_), where y_t_ is concentration at time t in perfumed flies and y_n_ is daily mean concentration in non-perfumed flies. R^2^ = .9837, slope = .0716, implied loss rate per day is 329%. Proportional loss rate is constant over time.

To more directly estimate θ we applied excess quantities of several compounds to groups of flies at hour zero and measured remaining quantity at 4 time points after application (see Methods). Fitting a standard geometric loss rate (constant proportional loss), we found turnover values of 146%/day for 7-C23∶1 and 329%/day for 9-C25∶1 (Figure 14).

Turnover due to cyclic hourly mean change can be estimated from the fitted curves shown in Figure 13 by measuring peak-trough differences summed over the day (see Methods). Turnover rates in LD were 30% for Cluster 1, 74% for Cluster 2, 68% for Cluster 3, 135% for Cluster 4, and 160% for Cluster 5 (methyl-alkanes). Cyclic turnover estimates were lower in LD than in DD for members of Clusters 1 and 2 (long chain n-alkanes and alkenes) but higher in LD for Clusters 3, 4, and 5. The most dramatic LD-DD turnover change was for Cluster 4, (tricosenes, C21, cVA) which declined from 135% in LD to 49% in DD, due to loss of 3-hour cycles in DD. Summed over all compounds, cyclic turnover was estimated at 97% in LD and 51% in DD.

Turnover due to AV changes was estimated assuming no hourly changes in TA or cyclic mean abundance (see Methods). Estimates of turnover due to AV were 133%/day in LD and 119% in DD.

We relate the above turnover rate estimates to the total lipid content of the fly as a means of quantifying the metabolite cost of turnover. We measured total ether-extractable lipids in LD and DD males using the method of Clark [35] (see Methods). Lipids were 35.2 µg/fly±2.15 s.e.m in LD and 37.6 µg/fly±3.17 s.e.m in DD. The mean TA represents 6.4% of body lipids in LD and 6.1% in DD. Turnover in CHs thus represents from 1.9% (LD TA turnover rates) to 8.6%(LD AV turnover) of total lipids/day.

In this section we used several different approaches to estimate CH turnover rates. A stochastic null model of TA changes reproduces the lognormal distribution of TA well, and suggests a lower bound for TA-based turnover in the 30–50%/day range. The important term in the model is the loss rate, which we independently estimate by measuring the loss rate of synthesized CH applied to flies. This approach yields a turnover rate several times higher. Secondly we use the cyclic peaks and troughs of hourly compound abundance to estimate the turnover rate due to cyclic change, finding rates which are chemical cluster dependent and which vary between LD and DD, but are generally in the 50–130%/day range. Thirdly, we consider the consequences of the observed large changes in AV, even if there were neither changes in TA nor compound cycling. This provides us with a third set of AV-based turnover estimates, again in the 100–130%/day range. In summary, these estimates of turnover suggest that non-trivial proportions of CH are removed each day from the cuticle and must be replaced. These amounts are equal to 2–8% of total body lipids per day.

## Discussion

We have presented new methods for the analysis of CH variation, and new results found using these methods. We discuss each in turn below.

### Model-based Approach Reduces Variance of CH Estimates

Research on CH is complicated by the high variability in Total Abundance (TA). We found that TA is approximately lognormally distributed distributed and unaffected by light or time. The long tails of a lognormal distribution can result in flies in the same conditions having TA varying over a range of 4. This high variability increases standard errors for each compound measured, unless normalization is done.

Several normalization methods have been published. One method expresses compound abundances as a proportion of the total abundance (TA) for a fly. This method, which we call Relative Abundance (RA), succeeds at reducing error variances for many compounds and has the virtue of great simplicity. However, we show in Figure 2 that an assumption of the RA model (that the regression of compound abundance on TA passes through the origin) is violated in our data. When compound regressions have non-zero intercepts, RA values will depend systematically on TA (Figure 2b).

We generalize the RA model to allow non-zero intercepts (Equation 1) while retaining the simplicity of the linear model. We investigate the question of what value to use as the latent variable or factor **x**_i_ by using Factor Analysis to evaluate several alternatives. All of the 3 factor estimators we evaluated explained significantly more variance than RA normalization (Table 1) for the 12 most abundant compounds. We chose the simplest factor estimator, which was intermediate among the 3 evaluated in variance reduction, and normalize the data using Equation 3. This “FA normalization” produces smaller error variances than RA, is expressed in the same units as the original data (in our case µg), and does not change treatment means. FA normalized data is thus well suited for answering questions about the effect of treatments such as LD versus DD or time of day. In addition, it is simple to implement in widely accessible tools such as Excel; we have provided a worked example as a spreadsheet which can easily be adapted to new datasets (Excel S1).

FA-normalized data minimizes the large errors due to between-fly variations in total abundance, allowing us to detect common patterns of expression between compounds. Using multivariate clustering techniques we found 5 clusters of compounds as shown in Figure 4. Each cluster is centered around one “exemplar” member, for example C27 for Cluster 1, 7-C25∶1 for Cluster 2, 7-C23∶1 for Cluster 4, and 2-MeC26 for Cluster 5. Chemically similar compounds tend to be clustered together, but carbon chain length is another strong determinant of cluster membership. Interestingly, when we applied the same clustering methods to slopes fitted to equation 1, we found identical exemplars and highly similar clusters, showing that chemical similarities determine each of several components of patterns of variation. Again, for flies kept in the dark (DD) the same exemplars and similar cluster definitions were found, even though the DD treatment induces significant shifts in the patterns of expression of many compounds. The robustness of these clusters, and their clear relation to the chemical types of the compounds, shows that pathways of CH synthesis and the genes whose products control them may have a fairly direct relationship to observed patterns of CH abundance, in spite of the intervening complexities of hemolymph transport, cuticular deposition, and loss.

Equation 1 is applied within each treatment (condition, hour) to determine whether slopes and intercepts are affected by treatments. Expressing slopes in dimensionless units (using data scaled to means of 1; Equation 13) reveals variations of slope with strong chemical patterning. This dimensionless slope tells how much a given compound's abundance depends on the total abundance; it is formally identical to the beta value used in Mathematical Portfolio Analysis, which is commonly called “stock volatility” [19]. Expressing this as a percentage, we call this measure Abundance Variability, or AV. AV is affected by chemical groups, chain length, and double bond position, being highest in monoenes and lowest in n-alkanes, and generally decreasing with increasing chain length (Figure 3). Compounds with AV>100% increase more rapidly than expected as TA increases. The behaviorally important tricosenes and pentacosenes have AV>100%, and so will make up a larger proportion of total CH in flies with high TA. In the absence of light (DD), n-alkanes and methyl-alkanes generally show AV<100%, and so vary less with TA than expected.

Using our linear model, we derive in Equation 4 a simple expression based on both the slopes (AV) and intercepts which predicts the relation of the useful Desaturation Index (DI) [18], [33] to total abundance. DI varies between −1 and 1 and measures the balance between desaturated and saturated CHs, which is due (in males) to the activity of the desaturase enzyme encoded by the gene *desat1* [11], [33], [34], [36]. Equation 4 shows that DI depends hyperbolically on TA (Figure 12). Thus the generalized linear model of Equation 1 leads both to a powerful FA normalization technique and predicts shifts in DI based on total abundance. Any other Balanced Ratio (BR; [18]) of hydrocarbons will follow a similar curve and will be described by Equation 4, so long as Equation 1 holds for the constituent CH's of the ratio. Using the linear model, we were able to explain the occurrence of unusually large and small values of DI as being due to a sudden shift at CT 13 in LD of model slopes (AV values). Thus the general linear model provides insight into short term changes affecting the balance between desaturated and saturated compounds on the cuticle.

In each experimental comparison we have compartmentalized variation in CH into 3 measures: total abundance TA, FA-normalized mean abundance, and abundance variability AV. Each measure provides valid, but differing, insights into CH variation. For example, comparing wild-type males between DD and LD showed that mean TA responds little to presence/absence of light, but TA inherent variation σ*_TA_* does respond. The second measure, compound FA-normalized mean abundance, revealed LD-DD differences in only a few cases when 24-hour averages were used (Figure 6) but in almost all cases when hourly averages were used (Figure 9). The third measure AV in LD showed that methyl-alkanes have higher AV in LD (Figure 5), while AV for many monoenes is lower in LD. The interaction of TA variation and AV is predictive of variability in DI and other Balanced Ratio indices. Separating CH variation into TA, FA-normalized mean, and AV components is conceptually similar to compartmentalizing variation due to different ANOVA factors, and yields greater insights into patterns of CH abundance.

### Changes in CH Abundance affected by light, time and biosynthesis

Our results show that CH compounds vary in response to light and time of day. The patterns of variation are specific to groups or clusters of compounds. Clusters were determined from statistical analyses, but coincide with chemical categories. Both carbon chain length and bond type determine cluster membership. Some clusters are higher on average in day hours, others at night, but for many compounds there is considerable shorter term variation at characteristic frequencies. Variability in compounds and direct estimates of compound loss rates suggest that CH turnover is appreciable; the highest turnover rates are associated with day-peaking clusters, the lowest rates with night-peaking clusters.

Whether flies were in LD or DD treatments, the difference between “day” (CT 0–11) and “night” (CT 12–23) compound mean levels was very consistent (Figure 7). Long chain n-alkanes and monoenes are on average higher at night, while methyl-alkanes are on average higher in the day. Within day and night, strong hourly variations are found in both mean levels and AV values, with some of the largest fluctuations near the day-night boundaries (Figure 8). The timing of these fluctuations is shifted by the presence of a regular light cycle (LD) or its absence (DD) (Figure 9). Thus both time of day and light environment affect CH levels.

Our data shows that there are day-night differences in mean compound abundance for short-chain methyl-alkanes, long-chain alkenes, and n-alkanes, with methyl-alkanes high in day and long chain unbranched compounds higher at night. In addition, the abundant Cluster 4 monoenes such as 7-C23∶1, while not showing significant day-night differences, do show a significantly larger day-night difference in LD than in DD. It is thought that most chain length differences are due to the activity of genes such as *smoq* and *sept* [6] rather than by the final chain reduction steps [1]. Thus, one interpretation of our data would be that elongase activity is higher at night or in the dark than in day. The data of Roualt et al. show that higher raising temperatures increase pentacosenes at the expense of tricosenes, perhaps due to increased elongase activity[18]. Our flies are maintained in constant temperature, so the light signal is not associated with increased temperatures as it would be in the wild. If temperature-dependent elongase activity is an important determinant of long versus short chain compound balances over periods of 12 hours or less, then in a natural environment which is hotter in day than at night, the differences we observed between long and short chain compounds might be reduced by higher elongase activity during hotter daytime periods. In this scenario, the constant-temperature, day-night difference we observed might represent a compensatory mechanism which, in combination with heat-dependent elongase, tends to produce more even compound levels over day-night intervals. Alternatively, if differential synthesis of compounds is not the cause of day-night differences, then differential transport to the cuticle or differential loss from the cuticle must be invoked to explain day-night changes. Melting temperature increases with chain length, desaturation decreases melting points[37]. Hence the fact that compounds with lowest melting points are most abundant in day and those with highest melting points are most abundant at night in our constant-temperature regime, might represent over-provisioning in day of compounds more likely to be lost in higher day temperatures, if differential loss is important.

The question of whether temporal variation in CH is regulated by the endogenous circadian clock, rather than by environmental or other cues, cannot be fully addressed by this study, which covers only a 24 hour period in full darkness in one genotype. Proof of clock regulation requires that additional criteria be met. Nevertheless, our Fourier analysis of periodic variation in FA-normalized data demonstrates that many longer chain compounds show highly significant 24-hour cyclic components in both LD and DD (Figure 13), suggesting the clock may play a role in compound cycling. However, our analysis shows that shorter-chain compounds also have highly significant cyclic components with 3-hour periods in LD which are absent or diminished in DD. Such ultradian oscillations have been detected in *Drosophila* locomotor activity rhythms[45]. The very short period of these oscillations and their dependence on light suggests that other factors in addition to the circadian clock must play a role in regulating hourly CH variation.

Rapid changes in CH values imply that hydrocarbons are removed from the cuticle at some times in the day and therefore must be replaced at others. We use several independent methods to estimate CH turnover rates. The largest potential contributor would be changes in TA, if this in fact varies over short times on a single fly. Since our CH assay kills the fly, we cannot directly observe TA fluctuations over time. Accordingly we asked whether the observed distribution of TA is consistent with a stochastic model in which removal of CH (loss) is proportional to the quantity present on the cuticle, while replacement (gain) is approximately constant over time (Equation 5). This model robustly reproduces the observed lognormal distribution of TA, and indicates that TA-derived turnover may be as high as 31%/day in LD and 53%/day in DD. As a more direct test of this model, we applied excess CH to flies and monitored the decline back to normal levels; we observed loss rates in this experimental manipulation of 146%/day for 7-C23∶1 and 329%/day for 9-C25∶1. A third line of evidence is based on the hourly fluctuations of FA-normalized mean abundances; using our Fourier fits of cyclic compound abundances, we estimated the minimum cyclic turnover rate which was highest for Clusters 4 (short chain alkenes, 135%/day) and 5 (methyl-alkanes, 160%/day) and averaged 97% over all compounds in LD. The reduced short-term variability at 3 hour periods for Cluster 4 compounds in DD contributed to a reduced cyclic turnover rate estimate for DD of 51% over all compounds. Finally, a fourth line of evidence is derived from fluctuations in AV, yielding turnover estimates of 133%/day (LD) and 119%/day (DD).

Although further investigations are required to more precisely determine CH turnover rates, our data suggest that turnover is somewhere between 30% and 130%, with the modal estimate close to 100%/day. As CH levels in our flies represent around 6.4% of total body lipids, a 100% turnover rate per day represents an allocation of 6.4% of lipids to CH maintenance. Since about 50% of CH are monoenes which have been shown to be involved in male sexual signaling [1], [16], [38], this implies that the metabolic cost of male sexual signaling may be equivalent to the daily production of 3% of total body lipids – a small but significant cost, especially given the high energy cost of lipid production. A recent study using wild-derived *D. melanogaster* strains[39] showed that there is significant segregating genetic variation for levels of almost all male CHs, and that some QTLs for CH variation co-locate with QTLs for starvation and longevity. To Foley et al. this “suggests that a large proportion of variation in CHC expression may reflect variation in a number of biological processes that require the expenditure of energy or lipid resources”[39].

This study has identified strong roles for chemical compound clusters, light, and time of day in patterning cuticular hydrocarbon variation in male *D. melanogaster*. We have shown that CH of male wild-type *D. melanogaster* vary significantly throughout the day and night. Compounds which are members of the same cluster show similar patterns of variation. A linear model relating compound abundances to total abundances leads to a new method of compound normalization, which in its turn reveals cyclic variations in abundance whose frequencies depend on chemistry (compound chain length and type) and environment (LD vs DD). The methods developed here, as well as the findings, will enable us to pursue our working hypothesis that chemical signals emitted by individual flies are physiologically regulated by circadian clocks and socially regulated by other flies.

## Materials and Methods

### *Drosophila* stocks and culturing

All stocks were reared on standard sucrose-yeast-agar medium in a 12∶12 LD cycle at 23°C and 70% HR. The Canton-S strain was used as wild-type in all experiments. For all procedures male and female flies were anesthetized with CO_2_ and separated within 8 hrs of eclosion. For determining hydrocarbon composition and quantity as a function of time, flies were reared in groups of 40 males housed in plastic vials (92 mm×25 mm diameter) containing 10 ml of medium, and were tested 5–6 days post eclosion.

Canton-S stocks were obtained from J. Hall, Brandeis University.

### Hydrocarbon extraction

To assess the composition and/or level of cuticular hydrocarbons as a function of time, hydrocarbon extracts were obtained from Canton-S male flies every hour throughout a 24 hr period. Cuticular hydrocarbons were extracted from individual male flies as described [40]. In brief, hydrocarbon samples were obtained from three individuals per time point, selected from a single vial containing 40 flies. The flies were removed either under light or red-light conditions depending on light schedule, anaesthetized using ether, and sorted under a microscope. Each fly was placed into an individual glass micro-vial containing 50 µl of hexane containing 10 ng/µl of octadecane (C18) and 10 ng/µl of hexacosane (C26) as internal standards. To achieve efficient extraction of the cuticular hydrocarbons the micro-vials were gently agitated for 5 min on a vortex mixer. The flies were removed using a thin wire probe, and the extracts stored at −20°C prior to analysis. Experiments were repeated from 3 to 5 times per treatment.

### Gas Chromatography

GC analysis was carried out as described [40], supplementary material). A 0.5 µl sample of each hexane extract was injected on a FID Varian CP3800 gas chromatograph with a PTV injector (cool-on-column mode) fitted with DB-1 20 m×0.18 mm Agilent 100–2000 fused silica capillary column connected to a 5 m×0.25 mm deactivated silica retention gap (Agilent Technologies, Mississauga, Ontario, Canada). Carrier gas was Helium at a flow rate of 1 ml/min. The temperature program started at 50°C (isotherm 1 min) then increased to 150°C at 36.6°C/min and from 150°C to 280°C at 5°C/min (isotherm 8 min). The injector temperature was 50°C for 0.1 min and then ramped to 280°C at 200°C/min.

Compound identification was conducted on a Shimadzu GC-17A gas chromatograph fitted with a HP-5MS fused silica capillary column (0.25 mm×30 m, ID 0.25 µm) linked to a Shimadzu QP5050A mass spectrometer (Electron impact at 70 eV). The injector was used in splitless mode set to 0.5 min with helium as carrier gas (1 ml/min). Injector temperature was held constant at 280°C. The oven temperature was increased 1 min after injection from 60°C to 225°C at 6°C/min and from 225°C to 310°C at 3°C/min (isotherm 10 min). The pressure flow was increased from 57 kPa (1 min) to 185 kPa (1.83 min) at 2 kPa/min. Mass was scanned between 45 and 550 amu. The mass spectra were interpreted by fragmentation analysis and comparison to published criteria[40], supplementary material). Reported quantities are normalized to the internal C26 standard. Double bond positions of low abundance C22 and C24 compounds were not directly determined; however our compound designations align with those of other recent GC studies on *D. melanogaster* males [39].

### Lipid measurement

Total ether-extractable lipids were measured on 40 5-day old Canton S male flies kept under the same conditions as those used for CH measurements, at hour CT 3 in LD and in DD as described [35]. Briefly, flies were frozen in liquid nitrogen and stored at −20°C until testing. They were dried for 24 hours at 65°C and weighed in groups of 10 to the nearest 10 µg on a Mettler Toledo balance to establish dry weight, then soaked in diethyl ether for 24 hours, after which the ether was discarded and flies were dried overnight at 65°C and then reweighed. The change in weight is reported as total ether extractable lipids.

### CH Loss Rates

For perfuming experiments, 9-C25∶1 was synthesized and purified as previously described[41]. 31 µg of 9-C25∶1 was dissolved in hexane to facilitate an even distribution around a 2mL glass vial. Hexane was evaporated using nitrogen gas and then 6 male flies were added. The vial was vortexed for 10 seconds and rested for 10 seconds, this was repeated six times. Flies were transferred to normal food tubes and held under LD conditions for the stated number of hours, and then 5 flies were sampled for GC analysis as described above. All perfuming experiments began at CT 0.

### Statistical analysis

Analysis of variance, general linear model fits, Student t and F tests, multidimensional scaling, and hierarchical clustering were performed in version 2.4.1 of the *R* computing environment[42]. Non-hierarchical Affinity-Propagation clustering was done using the *apcluster.m* algorithm [30] in MATLAB 7.3.0. False Discovery Rate tests were implemented in *R* by the authors according to Theorem 1 of [32].

Each sample contains known spiked-in amounts of an internal C26 standard. Compounds whose mean abundance is less than 5 ng/fly have a high coefficient of variation; these include 2MeC22 and C28, which are omitted from most analyses. Some compounds are above the 5 ng/fly cutoff in some conditions but below in others; we omit them when they fall below – examples include 5-C25∶1 and 5-C24∶1.

For compounds above the 5 ng/fly cutoff in a treatment, we divided the absolute abundance of each compound for one fly by the total abundance of all compounds for that fly, giving a Relative Abundance (RA) value for each of 20–22 CHs.

Let Y = {y_i,j_} be the matrix of observed absolute abundances of compound j (j in 1,M) in fly i (i in 1,N). Define the total absolute abundance T_i_ for each fly as the sum of all compounds, and the mean-1 normalized abundances *y*′*_i,j_* and T′
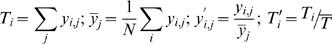
(6)Then from equation 1 and the RA model assumption that all intercept terms are 0, we find:
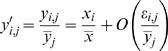
(7)

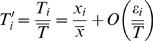
(8)

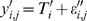
(9)Thus in the RA model mean-1 normalized compound abundances co-vary with normalized TA with slope 1 and a zero intercept. If the slope differs from 1 or the intercept differs from zero, the RA model is invalid.

Factor analysis to determine optimal estimators of the latent variable x in equation 1 was done using the *factanal* program of the *R* statistical language (version 2.4.1)[42] using Thompson's regression estimates of scores [43]. A single factor explained 50.5% of sample variance in LD and 51.6% in DD. Factor 1 loadings were high for compounds with 23 or fewer carbons and lowest for long-chain n-alkanes and methyl-alkanes. Four factors collectively explained 78% of variance with factor 2 loadings highest on pentacosenes, factor 3 highest on C23,C25, and C27, and factor 4 highest on methyl-alkanes. We evaluated several score estimators in order to find one which was simple to calculate (for those without access to factor analysis programs) but which explained significant variance. The three estimators evaluated were (a) scores from the above single factor analysis, (b) TA^20^, the sum of compound abundances, excluding 4 compounds with high uniquenesses (residual variance) on all factors (7-C22∶1, 2-MeC22, 5-C25∶1, and C28), (c) weighted mean total abundance R_i_:
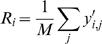
(10)No single estimator was superior for all compounds; (a) reduced variance most for short chain compounds, as expected from factor weightings, (c) explained more variance for long-chain n-alkanes and methyl-alkanes, while (b) TA^20^ represented a compromise with intermediate predictive power for most compounds, but much greater simplicity of calculation. In Table 1, the range of within-hour variance explained by the 3 score estimates is given in the FA columns, where it can be seen that any of the 3 estimators explains more variance than the RA method in each of the compound classes and LD/DD conditions. For simplicity, we use estimator (b), the total abundance TA^20^ of 20 compounds, as our score estimator in normalization and AV estimation. Note that method (c), weighted mean abundance, would be more appropriate in studies focused on methyl-alkanes and n-alkanes. This method allows less abundant compounds to contribute more to score estimates, which is also true of log-based methods such as logcontrast.

The FA normalization and AV calculations are performed in two steps. The first step is common to both calculations and estimates the intercepts and slope of equations 1 using linear regression on x_i_ = TA^20^:
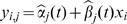
(11)In this step the regression is performed only on measurements taken during the same hour t, resulting (for our data) in 24 sets of slopes and intercept estimates. In our data, where there were a minimum of 10 replicates per hour, these regressions were usually highly significant (data not shown). In a study where measurements were performed at a single time, only a single slope and intercept would be calculated.

The second step in FA-normalization is given in equation 3; note that the mean score 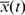 is the mean at hour t; in a study conducted a one time the mean of all scores would be used. Thus the expected value of the FA-normalized compound abundances is:
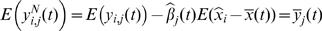
(12)That is, the FA-normalization does not change the mean compound abundances at hour t, only their deviations about the mean. If the regression of compound j at time t is not significant, we set 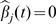.

To calculate the abundance variability of compound j at time t, we adjust the slope 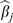 from equation 14 by adjusting for the mean of compound j and the mean of x:
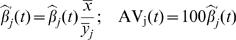
(13)Note that the mean abundance terms 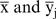 in equation 13 are independent of time t and are thus global means over all hours. This is done to avoid introducing a correlation of hourly AV with hourly mean compound abundance.

The FA-normalization minimizes variation due to TA changes. Since the first principal component axis is close to the TA score, by using FA-normalized data we are effectively focusing our attention on the 2^nd^ and higher principal components of the data. Just as in RA normalization, FA-normalization removes degrees of freedom, so multivariate tests on the full FA-normalized data matrix may encounter multicollinearity or singularity of the covariance matrix. The logcontrast method deals with this by discarding one variable; in the FA method four variables are excluded from the TA^20^ score estimator, avoiding the singularity issue in a similar way to logcontrast. We analysed the number of principal components explaining significant amounts of variation in non-normalized data and found that 6 were significant (data not shown). Thus rather than being a full 24-dimensional data set due to the 24 CH compounds, the data can be represented in 1 TA dimension and 5 additional dimensions. Note that our clustering method identifies 5 clusters.

To cluster compound abundance patterns, we calculate the Pearson correlation matrix **R**_FA_ of hourly FA-normalized compound abundances, and defined the distance **D**_FA_ = 1−**R**_FA_. Non-hierarchical Affinity-Propagation clustering was done using the *apcluster.m* algorithm [30] with the preference value p = 0.7. Multidimensional scaling of **D**_FA_ was performed using the R program *cmdscale* with dimensionality = 2; these MDS values, after rotation or reflection if required, give x-y coordinates for compounds which are then joined using Affinity Propagation cluster memberships (Figure 4 a,b). The x-y coordinates are related (before rotation and reflection) to the weighting of each compound on the first two Principal Component axes. To determine whether cluster relationships thus found were unique to normalized abundance patterns, we similarly analyzed the correlation matrix **R**_AV _of hourly compound AV values, leading to the cluster depictions in Figure 4 e,f. As an alternative check on cluster membership, we used the R program *hclust* using Ward's minimum hierarchical clustering method[31] applied to **D**_FA_ (Figure 4 c,d).

The DI index value can be related to fitted slope and intercept values by substituting equation 1 into the definition of DI:
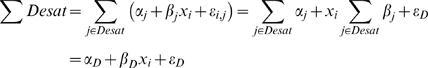
(14)

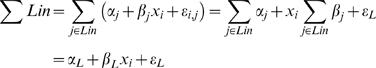
(15)

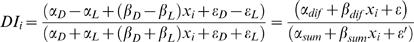
(16)Note that in this formulation, the scores x_i_ are adjusted to mean 0 by factor analysis convention; thus DI at mean x = 0 is dependent only on the intercept terms, but at high or low abundances DI will show a hyperbolic relationship to x and is influenced by the slope or AV terms. The groups *Desat* and *Lin* we used are identical to those of Marcillac et al and include the most abundant odd chain length alkenes and n-alkanes, respectively.

For detection of temporal pattern in FA-normalized hourly abundance data, we fitted the Fourier model:
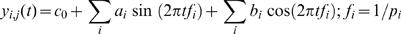
(17)for periods p_i_ which are integral harmonics of 24 hours (*p_i_*∈{2,3,4,6,8,12,24}). A stepwise regression model was used to eliminate non-significant periods; significance was determined by the F-test of variance explained by the terms of period p_i_ compared to residual variance, where the numerator degrees of freedom is 1 for p_i_ = 2 and 2 otherwise. Only periods with p<0.05 were retained.

Stochastic difference equations described by equation 5 (Random Coefficient Auto-Regressive, or RCAR) have a well defined stationary distribution with mean and variance given by [44]:
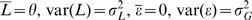
(18)

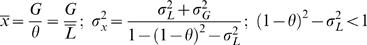
(19)We simulated equation 9 for 150 different combinations of distributions of L and G using one million time periods per combination and determined the following empirical formula for skewness:
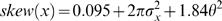
(20)This explained 93.3% of the variance in skewness in our 150 simulations. This equation can be solved for θ, given observed skewness and variance values for a distribution of data.

Cyclic turnover estimates were derived from the Fourier series stepwise regression of equation 17 by summing peak-to-trough heights of the fitted curve over 24 hours.

Turnover due to AV changes was estimated by constraining the hourly change in TA to be zero (i.e. assuming zero contribution from TA turnover) and asking how much individual compound abundances would have to shift to account for the change in AV and intercept values at the next hour. TA is the sum of the compound abundances in equation 1, that is:
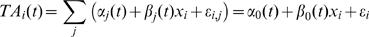
(21)If TA does not change for fly i from hour t to t+1, then equating (22) at t and t+1we find:
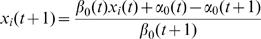
(22)The change from t to t+1 in compound j for fly i implied by (23) is thus:
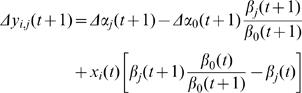
(23)To estimate turnover due to AV change, we calculated the above Δ*y_i_*_,*j*_ for each fly i and compound j. We then averaged positive Δ*y_i_*_,*j*_ values within an hour and summed these averages over 24 hours, to yield a compound-specific turnover amount. The compounds with largest AV turnover amounts were 7-C23∶1, 7-C25∶1, C23, and 2-MeC28.

## Supporting Information

Excel S1 This spreadsheet implements the FA normalization technique for analyzing a set of male *Drosophila* cuticular hydrocarbons. It is intended for GC-FID output. Sample data is included. New data can be copied into the file to apply the normalization.
(0.07 MB DOC)

## References

[1] Howard RW, Blomquist GJ (2005). Ecological, behavioral, and biochemical aspects of insect hydrocarbons.. Annu Rev Entomol.

[2] Jones WD, Cayirlioglu P, Grunwald Kadow I, Vosshall LB (2007). Two chemosensory receptors together mediate carbon dioxide detection in *Drosophila*.. Nature.

[3] Van der Goes van Naters W, Carlson JR (2007). Receptors and neurons for fly odors in *Drosophila*.. Curr Biol.

[4] Jallon JM (1984). A few chemical words exchanged by *Drosophila* during courtship and mating.. Behav Genet.

[5] Cobb M, Jallon J (1990). Pheromones, mate recognition and courtship stimulation in the *Drosophila melanogaster* species sub-group.. Animal Behaviour.

[6] Ferveur JF, Jallon JM (1996). Genetic control of male cuticular hydrocarbons in *Drosophila melanogaster*.. Genet Res.

[7] Levine JD, Funes P, Dowse HB, Hall JC (2002). Resetting the circadian clock by social experience in *Drosophila melanogaster*.. Science.

[8] Chertemps T, Duportets L, Labeur C, Ueda R, Takahashi K (2007). A female-biased expressed elongase involved in long-chain hydrocarbon biosynthesis and courtship behavior in *Drosophila melanogaster*.. PNAS.

[9] Chertemps T, Duportets L, Labeur C, Ueyama M, Wicker-Thomas C (2006). A female-specific desaturase gene responsible for diene hydrocarbon biosynthesis and courtship behaviour in *Drosophila melanogaster*.. Insect Mol Biol.

[10] Chertemps T, Duportets L, Labeur C, Wicker-Thomas C (2005). A new elongase selectively expressed in *Drosophila* male reproductive system.. Biochem Biophys Res Commun.

[11] Dallerac R, Labeur C, Jallon JM, Knipple DC, Roelofs WL (2000). A delta 9 desaturase gene with a different substrate specificity is responsible for the cuticular diene hydrocarbon polymorphism in *Drosophila melanogaster*.. Proc Natl Acad Sci U S A.

[12] Ferveur JF (1991). Genetic control of pheromones in *Drosophila simulans*. I. ngbo, a locus on the second chromosome.. Genetics.

[13] Takahashi A, Tsaur SC, Coyne JA, Wu CI (2001). The nucleotide changes governing cuticular hydrocarbon variation and their evolution in *Drosophila melanogaster*.. Proc Natl Acad Sci U S A.

[14] Coyne JA (1996). Genetics of a difference in male cuticular hydrocarbons between two sibling species, *Drosophila simulans* and *D. sechellia*.. Genetics.

[15] Coyne JA, Crittenden AP, Mah K (1994). Genetics of a pheromonal difference contributing to reproductive isolation in *Drosophila*.. Science.

[16] Mas F, Jallon JM (2005). Sexual isolation and cuticular hydrocarbon differences between *Drosophila santomea* and *Drosophila yakuba*.. J Chem Ecol.

[17] Gleason JM, Jallon JM, Rouault JD, Ritchie MG (2005). Quantitative trait loci for cuticular hydrocarbons associated with sexual isolation between *Drosophila simulans* and *D. sechellia*.. Genetics.

[18] Rouault JD, Marican C, Wicker-Thomas C, Jallon JM (2004). Relations between cuticular hydrocarbon (HC) polymorphism, resistance against desiccation and breeding temperature; a model for HC evolution in *D. melanogaster* and *D. simulans*.. Genetica.

[19] Markowitz H (1970). Portofolio selection: Efficient diversification of investments.

[20] Rouault J, Capy P, Jallon JM (2000). Variations of male cuticular hydrocarbons with geoclimatic variables: An adaptative mechanism in *Drosophila melanogaster*?. Genetica.

[21] Jallon J, Kunesch G, Bricard L, Pennanec'h M (1997). Incorporation of fatty acids into cuticular hydrocarbons of male and female *Drosophila melanogaster*.. J Insect Physiol.

[22] Skroblin A, Blows MW (2006). Measuring natural and sexual selection on breeding values of male display traits in *Drosophila serrata*.. J Evol Biol.

[23] Blows MW, Chenoweth SF, Hine E (2004). Orientation of the genetic variance-covariance matrix and the fitness surface for multiple male sexually selected traits.. Am Nat.

[24] Blows MW, Allan RA (1998). Levels of mate recognition within and between two *Drosophila* species and their hybrids.. Am Nat.

[25] Loehlin JC (2004). Latent variable models : An introduction to factor, path, and structural analysis.

[26] Bartholomew DJ, Knott M (1999). Latent variable models and factor analysis.

[27] Markowitz H (1952). Portfolio selection.. The Journal of Finance.

[28] Soroker V, Hefetz A (2000). Hydrocarbon site of synthesis and circulation in the desert ant *Cataglyphis niger*.. Journal of Insect Physiology.

[29] Cox TF (1994). Multidimensional scaling.

[30] Frey BJ, Dueck D (2007). Clustering by passing messages between data points.. Science.

[31] Ward JH (1963). Hierarchical grouping to optimize an objective function.. JASA.

[32] Benjamini Y, Hochsberg Y (1995). Controlling the false discovery rate: A practical and powerful approach to multiple testing. Journal of the Royal Statistical Society.. Series B (Methodological).

[33] Marcillac F, Bousquet F, Alabouvette J, Savarit F, Ferveur JF (2005). A mutation with major effects on *Drosophila melanogaster* sex pheromones.. Genetics.

[34] Ueyama M, Chertemps T, Labeur C, Wicker-Thomas C (2005). Mutations in the desat1 gene reduces the production of courtship stimulatory pheromones through a marked effect on fatty acids in *Drosophila melanogaster*.. Insect Biochem Mol Biol.

[35] Clark AG (1989). Causes and consequences of variation in energy storage in *Drosophila melanogaster*.. Genetics.

[36] Labeur C, Dallerac R, Wicker-Thomas C (2002). Involvement of desat1 gene in the control of *Drosophila melanogaster* pheromone biosynthesis.. Genetica.

[37] Gibbs A, Pomonis JG (1995). Physical properties of insect cuticular hydrocarbons: The effects of chain length, methyl-branching and unsaturation.. Comparative Biochemistry and Physiology – Part B: Biochemistry and Molecular Biology.

[38] Grillet M, Dartevelle L, Ferveur JF (2006). A *Drosophila* male pheromone affects female sexual receptivity.. Proc Biol Sci.

[39] Foley B, Chenoweth SF, Nuzhdin SV, Blows MW (2007). Natural genetic variation in cuticular hydrocarbon expression in male and female drosophila melanogaster.. Genetics.

[40] Ejima A, Smith BP, Lucas C, Levine JD, Griffith LC (2005). Sequential learning of pheromonal cues modulates memory consolidation in trainer-specific associative courtship conditioning.. Curr Biol.

[41] Ginzel MD, Millar JG, Hanks LM (2003). (Z)-9-pentacosene: Contact sex pheromone of the locust borer, *Megacyllene robiniae*.. Chemoecology.

[42] R Development Core Team (2006). R: A language and environment for statistical computing. 2.4.1..

[43] Thomson GH (1948). The factorial analysis of human ability.

[44] Nicholls DF, Quinn BG (1982). Random coefficient autoregressive models : An introduction.

[45] Dowse HB, Ringo JM (1987). Further evidence that the circadian clock in *Drosophila* is a population of coupled ultradian oscillators.. J Biol Rhythms.

