# Supplementary material for: A Model-Based Analysis of Chemical and Temporal Patterns of Cuticular Hydrocarbons in Male Drosophila melanogaster
Source: PLoS One. 2007 Sep 26;2(9):e962. doi: 10.1371/journal.pone.0000962 (PMC1978534; doi:10.1371/journal.pone.0000962)
Supplement: Supplementary file 1 [file pone.0000962.s001.doc]

## Sheet: GC Data

# replicates	Compound mean	0.04222941583333333	0.142417376	0.012380575066666665	0.062930615	0.00046938670000000004	0.08645837216666666	0.9665884038666664	0.08555156093333333	0.2745770121333333	0.04172833053333333	0.011418236633333334	0.016856453966666667	0.016947331566666664	0.019071879899999998	0.05331382666666668	0.3041778324666667	0.007397500166666666	0.0518775096	0.08980903023333332	0.0182465333	0.05102411373333334	0.006356073	0.13133584453333333	0.030345730133333335	2.366868608266667
15	Compound s.e.m.	0.004077119036112815	0.04967952529181957	0.0019634952236018293	0.005708943492958667	0.0001901132731143575	0.010410811706579525	0.09546671391838223	0.008898802157181666	0.021985818900498368	0.005459033285072676	0.0015417830059666678	0.001994826556416656	0.0013643088890635802	0.003633605179450565	0.00869015447877067	0.031165877712690714	0.0026923029434900944	0.00396950025483623	0.009509598172788519	0.0027479284047901968	0.004172577017430151	0.0013309567667187862	0.011384999857736805	0.002534820386723394	0.21148912984115265
	Compound (ug)/ replicate	nC21	7-nC22:1	CVA	nC22	2-MeC22	9-nC23:1	7-nC23:1	5-nC23:1	nC23	9-nC24:1	7-nC24:1	5-nC24:1	nC24	2-MeC24	9-nC25:1	7-nC25:1	5-nC25:1	nC25	2-MeC26	7-nC27:1	nC27	nC28	2-MeC28	nC29	TA20
	1	0.066653358	0.031632359	0.0233414365	0.0883194265	0.0013342295	0.150922084	1.5569422895	0.16394093	0.487789778	0.066509322	0.022459531	0.0229295435	0.028099683	0.0054102	0.14422314	0.5785426575	0.0209736845	0.0963500735	0.188871818	0.0385284055	0.0956071505	0.0124325935	0.262593055	0.045366332	4.133400214000001
	2	0.051112182	0.2967672735	0.0091200055	0.044951876	0	0.0843801265	0.7687246905	0.072145553	0.23763637	0.036956098	0.012641819	0.01197646	0.0171674065	0.019478955	0.061924264	0.342252584	0.033766964	0.052701013	0.111424671	0.030686811	0.049781464	0.007972835	0.1317009095	0.0211423525	2.167905611
	3	0.0527824775	0.014393706	0.0189276815	0.0697765515	0.002191977	0.132297909	1.2011785005	0.120675429	0.3609177965	0.0565746815	0.017931707	0.0200611755	0.021157164	0.0040130685	0.1063817375	0.45421018	0.023090771	0.069601527	0.137777852	0.033025512	0.0703266295	0.0086845635	0.191868848	0.034108999	3.1735954269999995
	4	0.037798218	0	0.00797643	0.055044553	0	0.0551882725	0.6874101755	0.0607214715	0.260060362	0.027450417	0.007401552	0.0107070995	0.015377982	0.0171026155	0.0283845935	0.161756252	0	0.052601322	0.06654211	0.005820638	0.0562661685	0.0056769185	0.1046996265	0.041175625	1.759485484
	5	0.0282748745	0.201682176	0.006710809	0.0488546885	0	0.053686471	0.705261274	0.0614710095	0.233178239	0.0294380815	0.00948461	0.0115425915	0.013242663	0.0111846815	0.0345382965	0.2613636365	0	0.0478704365	0.0542233355	0.0136005725	0.0579813885	0.009753042	0.0985146745	0.049749463	1.8301717964999997
	6	0.032716637	0.272071827	0.006529724	0.0560467965	0	0.051693647	0.6678002995	0.061420215	0.246905183	0.0274792545	0.007481975	0.0106108015	0.0162562915	0.0137396275	0.0324445655	0.218609713	0.0057815265	0.050469324	0.070398585	0.0136716095	0.05659094	0.0104067475	0.1199156575	0.0387022175	1.7994830639999997
	7	0.0352537265	0.148216379	0.00736895	0.053341149	0	0.043795009	0.634232122	0.05501591	0.176519846	0.0203483505	0.004940546	0.01214202	0.0107184725	0.013816781	0.018422375	0.1596884945	0	0.029810752	0.0566906715	0.011220901	0.03466756	0	0.1000669905	0.021018255	1.4990788819999998
	8	0.041252615	0.0126830545	0.014578975	0.060342573	0	0.0686453975	1.0058185145	0.076621339	0.2432662135	0.0309884935	0.0131406905	0.0194822175	0.013663703	0.0149058575	0.0351072175	0.3309361925	0.0068645395	0.045240586	0.0868200835	0.036087866	0.0575967575	0.005033996	0.1495162135	0.0363493725	2.3803608785
	9	0.0257162985	0.0565993425	0	0.0416862375	0	0.0342883985	0.5091592295	0.0466181305	0.15112729	0.026068577	0	0.009863786	0.0088069515	0	0.0187881635	0.1493658995	0	0.0355800845	0.0474401125	0.0147956785	0.0395725695	0	0.08677783	0.0241897605	1.2698449975000001
	10	0.032342494	0.0758085625	0.013740886	0.0511310525	0	0.095718826	0.926715274	0.0724434475	0.277341559	0.03140774	0.0082258365	0.0109366235	0.0133669845	0.056646102	0.045896429	0.2190128995	0	0.056833053	0.105159843	0.0084127875	0.047018134	0	0.1263787625	0.025425313	2.224154047
	11	0.024661017	0.733983051	0.0065254235	0.039661017	0	0.0584745765	0.689576271	0.0634745765	0.220084746	0.023135593	0.008644068	0.0115254235	0.0127118645	0.026355932	0.0409322035	0.275	0	0.04220339	0.0861864405	0.0116101695	0.0348305085	0	0.130084746	0.0261864405	1.8318644069999999
	12	0.0269049085	0.034709386	0.008626001	0.046621483	0	0.078250154	0.8395974535	0.0751694395	0.2356746765	0.0354282195	0.012117478	0.0151981925	0.0182789075	0.02721298	0.049804888	0.33056069	0	0.042924625	0.0848223455	0.0134524545	0.034093243	0	0.120045184	0.019408503	2.1141918265
	13	0.072237313	0.035617273	0.025614988	0.1033357875	0.001345485	0.1387626615	1.63735371	0.118605773	0.371366556	0.075220228	0.0162092865	0.0293214185	0.020017263	0.0366961995	0.064012084	0.365540352	0.0064608665	0.052461222	0.1076768805	0.014241832	0.040199538	0.0097991925	0.123099185	0.022263969	3.4342362470000003
	14	0.0404417845	0.2149895835	0.012367316	0.0743527	0.0013557755	0.096425383	1.1116695875	0.0966568565	0.2726265665	0.048559902	0.01023445	0.0212294565	0.020584637	0.0171951985	0.050940776	0.2824146025	0.004877484	0.0528752355	0.0658873715	0.0116894285	0.044856321	0.013127873	0.102989319	0.022072683	2.4560695750000012
	15	0.0652933335	0.0071066665	0.02428	0.1104933335	0.0008133335	0.1543466665	1.5573866665	0.1382933335	0.34416	0.09036	0.02036	0.03532	0.02476	0.02232	0.0679066665	0.4334133335	0.0091466665	0.05064	0.0772133335	0.0168533335	0.0459733335	0.0124533335	0.1217866665	0.0280266665	3.429186667

## Sheet: AV and slopes analysis

	Slopes beta-j	0.01710660498149983	0	0.008830603010062778	0.02285999324509659	0.0006969554213406089	0.04749923680906267	0.4348106416217826	0.04140098478857916	0.09966441152330914	0.023068210605409135	0.006884807574419361	0.007839809026021336	0.005857610924524099	0	0.0364043272196048	0.13560257690758054	0.006688136322918715	0.014721179339679457	0.03549333192470333	0.007224838525581953	0.01143313742852242	0.004016146895405468	0.04044910577787568	0
	AV	0.9587886909098685	0	1.6881992107827073	0.8597818469914393	3.5143771822515535	1.300330433046707	1.0647134334303965	1.1453992209887762	0.8591125861670559	1.3084497470420509	1.4271411116387613	1.1008126569911636	0.8180754216177113	0	1.6161709764311984	1.0551507974131893	2.139903961321217	0.6716416713987968	0.9354076412706792	0.9371776668390788	0.5303518688241321	1.4955290810729887	0.728953463072401	0
	AV s.e.	0.13817563825592952	0	0.15197961481014677	0.1497537327998933	0.7939939224116768	0.09813199763257763	0.08235253961052368	0.0576366854407308	0.0706838595009261	0.18219789952233253	0.13780459279293703	0.2042423344462282	0.10467955350273597	0	0.23463830876981895	0.12450071233011185	0.961207264829736	0.1473363820352449	0.2017808446510627	0.3885256068621791	0.20686561954370203	0.5004067002712761	0.17754841963069798	0
		nC21	7-nC22:1	CVA	nC22	2-MeC22	9-nC23:1	7-nC23:1	5-nC23:1	nC23	9-nC24:1	7-nC24:1	5-nC24:1	nC24	2-MeC24	9-nC25:1	7-nC25:1	5-nC25:1	nC25	2-MeC26	7-nC27:1	nC27	nC28	2-MeC28	nC29
	Compound	nC21		CVA		2-MeC22		7-nC23:1		nC23		7-nC24:1		nC24		9-nC25:1		5-nC25:1		2-MeC26		nC27		2-MeC28
	Slope	0.01710660498149983	0.001740329508603193	0.008830603010062778	-0.008520301989916062	0.0006969554213406089	-0.0011802152081323554	0.4348106416217826	-0.06255125432821862	0.09966441152330914	0.038684445137442236	0.006884807574419361	-0.004877198288516427	0.005857610924524099	0.0030831361499706854	0.0364043272196048	-0.03285043263448368	0.006688136322918715	-0.008432439743857696	0.03549333192470333	0.005800977097963886	0.01143313742852242	0.02396337965976494	0.04044910577787568	0.035598125835221525
	s.e. slope	0.002465314916749477	0.006152555869275341	0.000794972320469474	0.0019839703167272606	0.000157461291158788	0.0003929678048514894	0.03363135982220697	0.08393200351809549	0.008199932552246669	0.020464137384325044	0.0006647962815401949	0.0016590968707784726	0.000749530031056804	0.001870562401762844	0.005285238935284635	0.013190090893005558	0.003004193336691575	0.0074974062017480955	0.007656420772357934	0.019107723820826324	0.004459535633811197	0.011129426894566502	0.009852034691031384	0.02458720119315656
		0.7874028309149763	0.007555619889574743	0.9046844474612216	0.002436406251952647	0.6011200639795378	0.00048258243003133396	0.9278386980290715	0.10307233751749285	0.9191174712773313	0.02513089628590528	0.891893923107447	0.00203744680778681	0.8245020241211194	0.0022971361476014827	0.7849231283011541	0.016198034640233106	0.2760186846578831	0.009207157581621602	0.6230821491142454	0.02346515841751157	0.3358135571261178	0.013667445040862177	0.5645831227815999	0.030194207141083645
	F	48.14850943664696	13	123.38907453965646	13	19.591260743014942	13	167.15196019103527	13	147.7269234196993	13	107.25225938890321	13	61.07492841382917	13	47.443505139886945	13	4.95626451196031	13	21.490274125913864	13	6.572817451301819	13	16.8564448926479	13
	SS	0.0027486728283684482	0.0007421360949045869	0.0007324468530333096	0.00007716898051920128	0.000004562526465919085	0.0000030275154230743152	1.7758068400414253	0.13811078789715955	0.09329870354662911	0.00821030532572806	0.0004452244524431102	0.00005396546342928861	0.00032228228815533197	0.00006859884824802595	0.012448052580948107	0.0034108922406804916	0.0004201512197737822	0.001102032759526558	0.011832838481393675	0.0071579775742658825	0.0012277960816562878	0.0024283877012848455	0.015367854686317423	0.011851971883422664
	p(F)	0.0000009765123328454705		0.000000003513296250431661		0.00011932155270257752		0.0000000005321313345786247		0.0000000011505236474695528		0.000000008321404113884186		0.0000002456577417827231		0.0000010625081285132283		0.025127952783757988		0.0000755771188101266		0.010654597289742608		0.0002450716157939904
	Significant slope	0.01710660498149983		0.008830603010062778		0.0006969554213406089		0.4348106416217826		0.09966441152330914		0.006884807574419361		0.005857610924524099		0.0364043272196048		0.006688136322918715		0.03549333192470333		0.01143313742852242		0.04044910577787568
		Compound	7-nC22:1		nC22		9-nC23:1		5-nC23:1		9-nC24:1		5-nC24:1		2-MeC24		7-nC25:1		nC25		7-nC27:1		nC28		nC29
		Slope	-0.08434499092716193	0.3420508872900364	0.02285999324509659	0.00882401460299282	0.04749923680906267	-0.02596608035332834	0.04140098478857916	-0.01243913031408049	0.023068210605409135	-0.012871092997493755	0.007839809026021336	-0.0016993439118289065	0.0008832470869819657	0.016981350096479407	0.13560257690758054	-0.01677565001595216	0.014721179339679457	0.01703441234424887	0.007224838525581953	0.0011462897940044438	0.004016146895405468	-0.003149619012922834	0.001965341679702776	0.02569402460712675
		s.e. slope	0.060805839311788214	0.1517499127605257	0.003981672016236051	0.009936847972883908	0.0035846234738772394	0.008945954954276025	0.002083304662225888	0.005199193109157295	0.0032121826058248027	0.00801647400515845	0.0014545807471580165	0.0036301201329125747	0.0047588648596621165	0.011876446990393823	0.01600019396296911	0.039930836668203656	0.0032293489155953707	0.00805931505528764	0.0029952002399935237	0.0074749626066127894	0.001343809920628683	0.003353675247807948	0.0032792074195316695	0.00818374428294632
			0.1289257948864472	0.18635583056147115	0.7171612365167567	0.012202903602798989	0.931065384373598	0.01098604167437309	0.9681314780392795	0.006384846834380009	0.7986793052703718	0.009844596574913843	0.6908389023989867	0.004457953484786552	0.002642804613917853	0.014584819870748885	0.8467462100732727	0.04903689298365934	0.6151621970270543	0.009897207343076524	0.30918724122737185	0.009179595820720787	0.40725589748176316	0.00411846652739079	0.0268879596007751	0.010050012123287821
		F	1.924101670885004	13	32.9625824971837	13	175.58449970120668	13	394.92604112682545	13	51.573590000068684	13	29.04927496012808	13	0.03444749798755152	13	71.82661346395072	13	20.780465171489165	13	5.818413289148263	13	8.931892607231855	13	0.3592016749342389	13
		SS	0.06682115638098896	0.45147044259532465	0.0049084863868073	0.001935841132409659	0.021191839630145343	0.0015690104517238099	0.01609966126657845	0.0005299614982804014	0.004998310263185115	0.00125990906239647	0.0005773063873961113	0.00025835354054276735	0.000007327567418803898	0.00276532061860849	0.1727154867011216	0.03126001935538116	0.0020355445058664046	0.001273411271494022	0.0004902884761357584	0.0010954447326133288	0.00015150067713955195	0.00022050296498409862	0.00003628035470217461	0.0013130356678170186
		p(F)	0.1853674262378047		0.000008104590011249705		0.00000000039098713385516804		0.0000000000022935108154887296		0.0000006577720815198807		0.00001597853502580181		0.9662269446915481		0.00000009408622521574642		0.00008931004415949595		0.01567944940183991		0.0036241992658730604		0.7049504608241923
		Significant slope	0		0.02285999324509659		0.04749923680906267		0.04140098478857916		0.023068210605409135		0.007839809026021336		0		0.13560257690758054		0.014721179339679457		0.007224838525581953		0.004016146895405468		0

## Sheet: FA normalized data

	Slopes beta-j	0.01710660498149983	0	0.008830603010062778	0.02285999324509659	0.0006969554213406089	0.04749923680906267	0.4348106416217826	0.04140098478857916	0.09966441152330914	0.023068210605409135	0.006884807574419361	0.007839809026021336	0.005857610924524099	0	0.0364043272196048	0.13560257690758054	0.006688136322918715	0.014721179339679457	0.03549333192470333	0.007224838525581953	0.01143313742852242	0.004016146895405468	0.04044910577787568	0
	Mean FA norm. data	0.042229415833333346	0.142417376	0.012380575066666668	0.06293061500000001	0.001926935562284506	0.0864583721666667	0.9665884038666668	0.08555156093333334	0.2745770121333334	0.04172833053333334	0.011418236633333335	0.016856453966666674	0.016947331566666668	0.019071879899999998	0.05331382666666669	0.30417783246666663	0.021384457136630137	0.0518775096	0.08980903023333334	0.018246533300000003	0.05102411373333334	0.014755075517127876	0.13133584453333333	0.030345730133333335
	s.e.m. FA norm.	0.0018798886680618965	0.04967952529181957	0.0006061941403591101	0.003036163880081689	0.00022203484582259658	0.002733400508806154	0.025645085660958453	0.0015885925161247694	0.006252734763867201	0.002449401348042571	0.0005069303672915311	0.0011091685872605431	0.0005715428086621831	0.003633605179450565	0.004030179152747483	0.01220070633306239	0.002659078591403525	0.002462491258378519	0.005838288062138112	0.0022839447210111913	0.0034005515668048916	0.0014267160833040535	0.007512520306104459	0.002534820386723394
	% reduction in s.e.m. due to FA norm	0.5389173945104605	0	0.691267830411571	0.468174122965755	-0.16790817487549925	0.737445975794695	0.7313714423764149	0.8214824323470642	0.7156014614618031	0.5513122525300811	0.671205114254262	0.44397743067298884	0.5810752145326323	0	0.5362361897486543	0.6085235767932737	0.012340495398894341	0.37964703355835555	0.38606364264220655	0.16884853439783504	0.18502365502188922	-0.07194772886676339	0.34013874396324734	0
	Compound (ug)/ replicate	nC21	7-nC22:1	CVA	nC22	2-MeC22	9-nC23:1	7-nC23:1	5-nC23:1	nC23	9-nC24:1	7-nC24:1	5-nC24:1	nC24	2-MeC24	9-nC25:1	7-nC25:1	5-nC25:1	nC25	2-MeC26	7-nC27:1	nC27	nC28	2-MeC28	nC29
	1	0.036433999633385256	0.031632359	0.007741897185040189	0.04793652592568636	0.00010303572041462207	0.06701318092857866	0.7888355485659312	0.09080478186248994	0.31172944507726097	0.025758598877831877	0.01029730082039595	0.009080273072619846	0.017752028167739325	0.0054102	0.07991374538110982	0.33899641957387383	0.009158880302110966	0.07034464492278766	0.12617172536222762	0.02576549989823966	0.07541015187982242	0.005337943075998436	0.19113843121973184	0.045366332
	2	0.0545157634001761	0.2967672735	0.01087696874255414	0.04950016877354018	0.0016496019081323554	0.0938307170234103	0.8552359190005124	0.08038281702332739	0.25746590003749614	0.04154581832363091	0.014011640950610728	0.013536291901815474	0.018332854326365285	0.019478955	0.06916737805708907	0.36923247913861595	0.04959690391052436	0.055629982964722755	0.11848653070271965	0.03212428652781747	0.05205623529094053	0.017478527012922834	0.13974878482232259	0.0211423525
	3	0.038982120483946864	0.014393706	0.011803797226195067	0.05133478187311776	0.0038415789081323553	0.09397900079676366	0.8504050948330601	0.08727614424908245	0.2805158428508711	0.03796493734442777	0.01237754808789752	0.013736591304960941	0.016431672173481057	0.0040130685	0.07701339041400096	0.34481594451930553	0.03892071091052436	0.057725556823297525	0.10914442925013786	0.02719704100039525	0.06110321091414712	0.018190255512922835	0.1592374695752063	0.034108999
	4	0.0481884811792591	0	0.013339989245410563	0.06892932711792167	0.0016496019081323554	0.08403850735337076	0.9515068214726324	0.08586773098860397	0.3205948436492263	0.04146165882875486	0.011583267934525644	0.015468867199878854	0.018935796024076006	0.0171026155	0.05049596750346963	0.24411896882073725	0.015829939910524362	0.06154271790022442	0.08810016083506014	0.010208882995990147	0.06321046323150611	0.015182610512922835	0.12926773074115902	0.041175625
	5	0.03745593485372275	0.201682176	0.011450165481477828	0.06112357399165089	0.0016496019081323554	0.07917915995677385	0.9386227590806298	0.08369078604003072	0.2866678109111611	0.041818716585085104	0.013179664274817872	0.01575019200912519	0.016386424107761685	0.0111846815	0.0540763828532724	0.33414110719364276	0.015829939910524362	0.0557712465170513	0.07327249358296434	0.017478120302208824	0.06411751690637814	0.019258734012922836	0.12022358060979856	0.049749463
	6	0.042422677377983165	0.272071827	0.011540080495067339	0.06901722620930147	0.0016496019081323554	0.07864402732916134	0.9145055720495139	0.084910535287444	0.30345332937616987	0.04056782372960817	0.011388315292783203	0.015058995811175846	0.01957981526251348	0.0137396275	0.053099854513157324	0.29554865490267024	0.02161146641052436	0.05882190835189125	0.09053698845193528	0.017770878439076102	0.0630779369025578	0.019912439512922833	0.14286589539688	0.0387022175
	7	0.05009866255424774	0.148216379	0.015032056568871986	0.07317881628062023	0.0016496019081323554	0.08501435870841209	1.0115563296708006	0.09094325925685157	0.2630075983943409	0.040366706666729826	0.010915111280404052	0.018945325728674002	0.015801647080769407	0.013816781	0.050013676152823025	0.277363017595684	0.015829939910524362	0.04258564018950295	0.08749142029523026	0.017490541646435633	0.04458911919946666	0.009505692012922834	0.1351683089307142	0.021018255
	8	0.041021808062814726	0.0126830545	0.014459830117864949	0.06003413979360499	0.0016496019081323554	0.06800452496109505	0.9999519318229101	0.07606274572530639	0.24192151432708142	0.030677250968712384	0.013047798815701436	0.019376440678043196	0.013584670530484597	0.0149058575	0.03461604047949042	0.32910660588802665	0.02269447941052436	0.045041963870195684	0.08634119787419053	0.03599038652622066	0.05744249852019954	0.014539688012922835	0.14897046323414823	0.0363493725
	9	0.04448264806476399	0.0565993425	0.009687379999346065	0.06676418983183748	0.0016496019081323554	0.0863961827729389	0.9861567695716992	0.09203598832206297	0.2604615025942356	0.05137494869227185	0.007552796464723225	0.018464241605447032	0.0152328889868877	0	0.05872456999398211	0.2981251280484186	0.015829939910524362	0.05172956581395881	0.08637713564617785	0.022721496946540035	0.05211499120422919	0.009505692012922834	0.13115145407272802	0.0241897605
	10	0.03478385562469693	0.0758085625	0.01500114163430122	0.05439350640653294	0.0016496019081323554	0.10249765874170691	0.9887690839531307	0.07835197088011003	0.29156512176444965	0.034699909555758045	0.009208398792388685	0.012055478405563091	0.014202950873164293	0.056646102	0.05109185658735409	0.23836536176999484	0.015829939910524362	0.05893397965079028	0.11022525829352622	0.009443877160400942	0.04864980919201309	0.009505692012922834	0.13215143888471853	0.025425313
	11	0.03381312253451171	0.733983051	0.011249833210101663	0.05189120942705431	0.0016496019081323554	0.08388686774980886	0.9222017910231088	0.08562427729846724	0.2734056248817404	0.03547718258959817	0.012327468977226929	0.015719754266049754	0.01584571095400592	0.026355932	0.06040867150677506	0.34754794834814196	0.015829939910524362	0.05007928279432857	0.1051755221966686	0.015475488464659617	0.04094728505791868	0.009505692012922834	0.1517251875286433	0.0261864405
	12	0.031227350393679014	0.034709386	0.010857289349641705	0.052397672524378755	0.0016496019081323554	0.09025210829328677	0.9494640071028917	0.08563050709834892	0.2608575592603785	0.041257020716890476	0.013857109020987057	0.01717913021436034	0.019758989777250022	0.02721298	0.05900341624423042	0.3648243127322744	0.015829939910524362	0.046644325219360155	0.09379068638491013	0.015278003447427881	0.036982131370935076	0.009505692012922834	0.13026573387329313	0.019408503
	13	0.05397827643415265	0.035617273	0.016189488116557828	0.0789357704885213	0.0029950869081323554	0.08806351326547535	1.1732509021560322	0.07441570162497961	0.2649879884066183	0.05059796651630122	0.008860665696158638	0.020953460051775334	0.013765038658872136	0.0366961995	0.025155283215934812	0.22080254968000054	0.02229080641052436	0.03674831156883641	0.06979244661275097	0.006530273162719973	0.02799617709960433	0.019304884512922834	0.07992511847699402	0.022263969
	14	0.038915858798124946	0.2149895835	0.01157961767466411	0.07231356650301989	0.0030053774081323552	0.09218840515753604	1.0728840579213956	0.09296384863314668	0.26373640464321196	0.04650219531318935	0.009620318508588813	0.02053013795587418	0.02006213244278471	0.0171951985	0.04769347481873462	0.2703187215483125	0.02070742391052436	0.051562092071445804	0.06272133197972934	0.011044965919025852	0.04383647408858073	0.022633565012922834	0.09938121966111459	0.022072683
	15	0.04712067810453513	0.0071066665	0.01489909095290538	0.08620874985321188	0.0024629354081323555	0.10388736946168167	1.0954794697757528	0.09431231970974834	0.23828469582575823	0.06585422329121007	0.013046144582790277	0.02699162929463695	0.018537354133844396	0.02232	0.02923369227857639	0.2893602672403015	0.024976606410524363	0.03500142534160647	0.03950812603177131	0.009178257062841984	0.03382770514170065	0.021959025512922836	0.07881685097254786	0.0280266665
